# Early non-social behavioural indicators of autism spectrum disorder (ASD) in siblings at elevated likelihood for ASD: a systematic review

**DOI:** 10.1007/s00787-020-01487-7

**Published:** 2020-02-22

**Authors:** Daniela Canu, Sara Van der Paelt, Ricardo Canal-Bedia, Manuel Posada, Marleen Vanvuchelen, Herbert Roeyers

**Affiliations:** 1 Faculty of Rehabilitation Sciences, Rehabilitation Research Center (REVAL), Hasselt Unversity, Agoralaan, Building A, 3590 Diepenbeek, Hasselt, Belgium; 2 Research in Developmental Disorders Lab, Department of Experimental Clinical and Health Psychology, Ghent University, Ghent, Belgium; 3 Clinic for Child and Adolescent Psychiatry, Psychotherapy and Psychosomatics, Medical Center – University of Freiburg, Faculty of Medicine, University of Freiburg, Freiburg, Germany; 4 Instituto Universitario de Integración en la Comunidad (INICO), University of Salamanca, Salamanca, Spain; 5 Institute of Rare Diseases Research & CIBERER, Instituto de Salud Carlos III, Madrid, Spain

**Keywords:** Autism spectrum disorder, Siblings at elevated likelihood for ASD, Early signs, Non-social behaviours, Systematic review

## Abstract

We aimed at identifying early non-social behavioural indicators that predict later ASD. Likewise, we were interested in the moment in which non-social signs discriminate between children at elevated likelihood for ASD with a later diagnosis of ASD, and children at elevated likelihood for ASD with a typical developmental outcome. In addition, we intended to explore the developmental evolution of children’s symptomatology over time. A systematic literature search was conducted for longitudinal studies on early non-social behavioural indicators among siblings at elevated likelihood for ASD. The following databases were searched: PUBMED, Web of Science, PsycINFO, CINAHL and EMBASE. The study identification process was conducted by two reviewers independently. Compared to siblings at elevated likelihood for ASD with a typical developmental outcome, siblings at elevated likelihood for ASD with later ASD show impairments in attention disengagement, in gross and fine motor development and characteristic restricted and repetitive interests and behaviours, starting at 12 months of age. Moreover, early attention disengagement exerts a predictive role towards a later ASD diagnosis, given that from 12 months siblings at elevated likelihood for ASD who will receive an independent ASD diagnosis towards 24–36 months present marked difficulties in disengaging in comparison with siblings at elevated likelihood for ASD that will not satisfy the criteria for an ASD diagnosis. The findings call for a more comprehensive vision on early indicators of ASD. Further research is needed to extend results to other behavioural domains.

**Electronic supplementary material:**

The online version of this article (10.1007/s00787-020-01487-7) contains supplementary material, which is available to authorized users.

## Introduction

Autism spectrum disorder (ASD) is a neurodevelopmental disorder defined by a pattern of persistent impairments in social interaction and social communication across several contexts, together with narrow, stereotyped, repetitive behaviour [1]. Estimates of its prevalence vary from 1% [1, 2] to 1.5% [3] amongst the general population. The rate increases up to 18.7% [4] amongst younger siblings of children with ASD, considered at elevated likelihood for ASD (EL), suggesting a strong genetic contribution [5]. An early diagnosis of ASD can have a positive impact on children’s developmental outcome [6]. However, many of these children experience diagnostic delays [7]. Therefore, the average diagnostic age ranges between 38 and 120 months [8]. This finding is surprising, given that the majority of parents express their concerns before their child’s second birthday [9].

To shorten this temporal gap between parents’ first concerns and clinical diagnosis, a significant effort has been made. A vast majority of studies has identified some precursors of social interaction and communication deficits. Decreased frequency of orientation to social stimuli, complex babbling, word production, gesture use and imitation are among the most common early signs, which often appear late in the first year [10, 11]. However, clear discrepancies in the social domain become more obvious by 18–24 months of age [12]. The majority of cognitive theories coming from these results propose a single initial impairment in social information processing or in social orienting (i.e. theory of mind; [13], social orienting; [14]).

More recently, growing literature is emerging on non-social behavioural indicators, due to the inability of social theories to fully cover the complex variety of the spectrum. Hence, new theorizations on the development of ASD have been proposed. Among them the model of domain general impairment, the cascade models and the cumulative models are particularly promising. According to the model of domain general impairment, gaze abnormalities in ASD are not unique to the social domain. On the contrary, they indicate a more basic attention deficit that, in turn, affects socio-communicative development [15]. Although referring to attention, this concept can easily be generalized to any non-social feature. This model paves the way for the possibility that non-social features are visible early in children’s development and before the social impairments are clearly manifested [15, 16]. If this is the case, we could identify ASD in EL children at an earlier age. The cumulative models state that brain systems subserving social and non-social cognition contribute to ASD via separate pathways [15, 17]. Cascade effect models imply interactions between different factors during development [15]. Despite the differences, these models represent an attempt to better describe the variability and complexity of people with ASD.

The present review seeks to supplement the wide literature on social indicators with the necessary insight regarding the non-social impairments of young children with later ASD. To our knowledge, this is the first systematic review to selectively focus on early non-social behavioural indicators of ASD in EL siblings, through a systematic comparison with younger siblings of children with a typical development, considered at typical likelihood for ASD (TL). Previous attempts have combined behavioural and biological signs as well as pre-, peri- and post-natal indicators of ASD in populations at elevated risk for ASD [18, 19]. In other cases, they included the whole range of behavioural indicators [20–22]. Alternatively, some reviews have selectively focused on a single behavioural domain. Examples are Sacrey et al. [23] about attention disengagement in EL children, Downey and Rapport [24] about impaired motor activity in children who later develop ASD and Leekam et al. [25] about repetitive and restricted behaviours in children with ASD. The cited reviews recognize that non-social signs are systematically evident within the ASD spectrum. Sacrey et al. [23] identified attention disengagement as an early marker of ASD, being atypical in the first year of life. No link between disengagement and other attention components has been explored, nor have studies assessed disengagement in complex, ecological settings. Downey and Rapport [24] concluded that motor abnormality is an observable trend in children with ASD. Nevertheless, the authors did not report specific motor patterns or explore the developmental moment in which motor abnormalities appeared. According to Leekam et al. [25], restricted and repetitive behaviours in ASD versus other clinical conditions did not differ in their systematic form or pattern, but rather in their frequency. Children with ASD present restricted and repetitive interests across a wide range of behaviours, while in other disorders they seem to be more specific to a single domain (for example only obsessions and compulsions in Obsessive Compulsive Disorder, hoarding in Prader–Willi syndrome). Finally, the frequency of repetitive and restricted behaviours is affected by age and developmental level in ASD.

The aims of the present review were: to summarize the state of the art on early non-social behavioural indicators of ASD that are able to discriminate between EL and TL samples, as well as within the EL sample; to identify at what age EL subgroups differ in various non-social domains and to explore whether early non-social behaviours are good predictors for a later ASD diagnosis, thus clarifying the nature of this association and describing the developmental evolution over time.

## Methods

### Study design

**Fig. 1:**
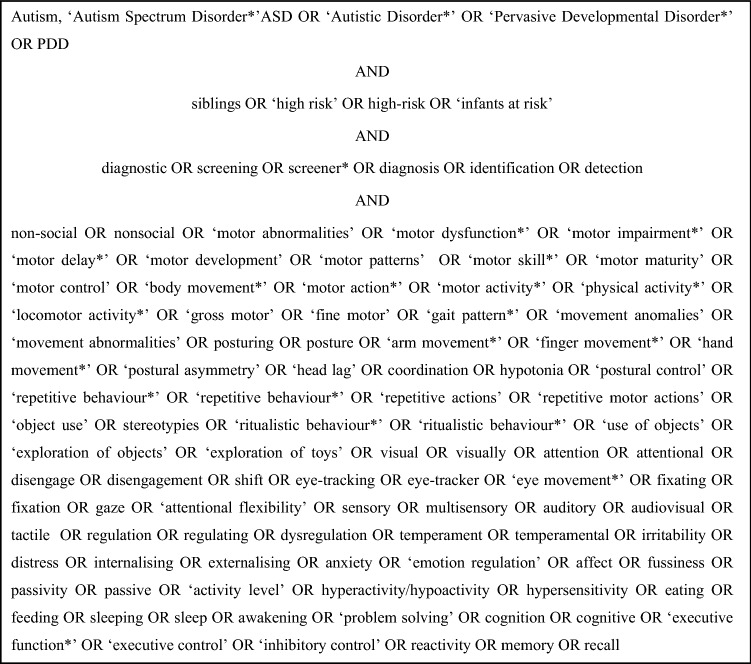
Keywords with mapped medical subject headings (MeSH) terms

A literature search was conducted using the databases PUBMED, Web of Science, PsycINFO, CINAHL and EMBASE. The search terms were defined through a combination of MeSH terms and terms chosen mutually by two reviewers (the first and second author of this review). Additionally, eligible studies were searched for by manually examining the reference list of other reviews. The search strategy is provided in Fig. 1.

### Study criteria

For each database, the search was limited to studies published in English between January 1, 2005 and April 15, 2019, thus covering the last 15 years of research on EL siblings since sufficient consensus on the core symptoms of ASD had been reached. The risk of bias was controlled already at a study level by defining strict inclusion and exclusion criteria prior to the beginning of the search process.

Eligible studies were longitudinal studies on EL children, more specifically younger siblings of children with ASD. We included studies on children aged 36 months at the latest, at the moment of their first assessment. Studies were selected if children were followed up until a diagnostic indication was made. Overall, across all studies EL participants were separated into three subgroups: children with ASD (EL-ASD), children with developmental delay (EL-Other) and children with a typical outcome (EL-TD). In two studies EL participants were stratified according to the severity of the autistic symptoms: EL-Autism, with higher ADOS scores (above autism cut-off), EL-ASD, with lower ADOS scores (above ASD cut-off, but below autism cut-off), and EL-TD with ADOS scores below ASD cut-off [26, 27].

In regard to our domain of interest, the search was restricted to non-social behaviours. With the non-social attribute we referred to observable behaviours (motor behaviour, repetitive/stereotyped behaviour, play and sensory behaviour) but also cognitive functions (attention, executive functioning), and personal characteristics (temperament), which do not activate any social competency and do not imply an interaction with another person.

As part of the non-social behavioural manifestations of ASD, we also included studies assessing participants’ attention towards social stimuli, provided that this cognitive competency was not assessed during social interaction. Examples are studies on preference towards social versus non-social stimuli, on the ability to recognize social stimuli (i.e. faces) and on free viewing of social scenes.

As for their contextual relevance, papers that explored the predictive role of non-social behavioural characteristics towards a later ASD diagnosis and studies assessing whether non-social behavioural features discriminated between clinical and control groups were included.

In line with the above, we excluded studies with premature infants, born before 32 weeks of gestation, as early prematurity is associated with increased risk for ASD [28]. Only one study [29] included participants (3 out of 50 TL participants) born between 32 and 34 weeks gestational age. Similarly, we excluded studies with infants that at the time of enrollment were affected by or had an older sibling or a first-degree relative with any known genetic or chromosomal syndrome or neurological disorder that could account for ASD or any other disorder known to often be in comorbidity with ASD (i.e. psychosis, schizophrenia, bipolar disorder). Such a decision was motivated by our confined interest in exploring early ASD indicators in EL infants, whose EL status comes uniquely from having an older sibling with ASD. Conversely, we did not aim at exploring early indicators of ASD, in which the ASD diagnosis is consequent to genetic, chromosomal or neurologic conditions, or due to a family member affected by another psychiatric disorder, all representing separate risk factors for ASD. We additionally excluded studies focused on biomarkers of ASD, given that the review focused selectively on the phenotypic characterization of ASD, but also studies that compared early indicators of ASD and other clinical conditions, as it was not a goal of the present review to identify atypical behaviours common to ASD and other disorders. Finally, studies in which the diagnostic indication was not mentioned were not considered in the final selection, since they prevented any conclusion to be reached.

### Data extraction and study quality evaluation

Information was extracted from each included article on: name of first author, publication date, type of study design, characteristics of participants (including age, presence of older sibling with ASD diagnosis, method for assessing ASD diagnosis), type of assessed behaviour, method for assessing behaviour, type of follow-up, type of outcome measures.

To ascertain the validity of eligible longitudinal studies the two reviewers worked independently. Additionally, several titles, abstracts and full texts were reviewed twice, whenever disagreement emerged. Disagreements were resolved by detailed discussion until a consensus evaluation on all articles was reached, guaranteeing a reliability above 90%.

Furthermore, all included studies were assessed for methodological features most relevant for the control of bias. The risk of bias was measured at a study level, and independently by the two reviewers, using the Newcastle–Ottawa Scale for evaluating the quality of nonrandomized studies (NOS; [30]). Three factors were taken into account: selection, indicating the representativeness of the exposed cohort (EL group) as well as the ascertainment that exposed and non-exposed (TL group) cohorts belonged to the same community; comparability, assessing whether confounding variables were adjusted for; outcome, based on the duration and completeness of the follow-up period, and ascertained through adequate assessment (expert clinical judgment using standardized diagnostic instruments).

In line with the guidelines of the NOS, “good” quality score required 3 or 4 stars in selection, 1 or 2 stars in comparability, and 2 or 3 stars in outcome, “fair” quality score required 2 stars in selection, 1 or 2 stars in comparability, and 2 or 3 stars in outcomes, “poor” quality score implied 0 or 1 star(s) in selection, or 0 stars in comparability, or 0 or 1 star(s) in outcomes.

### Articles’ identification process

**Fig. 2:**
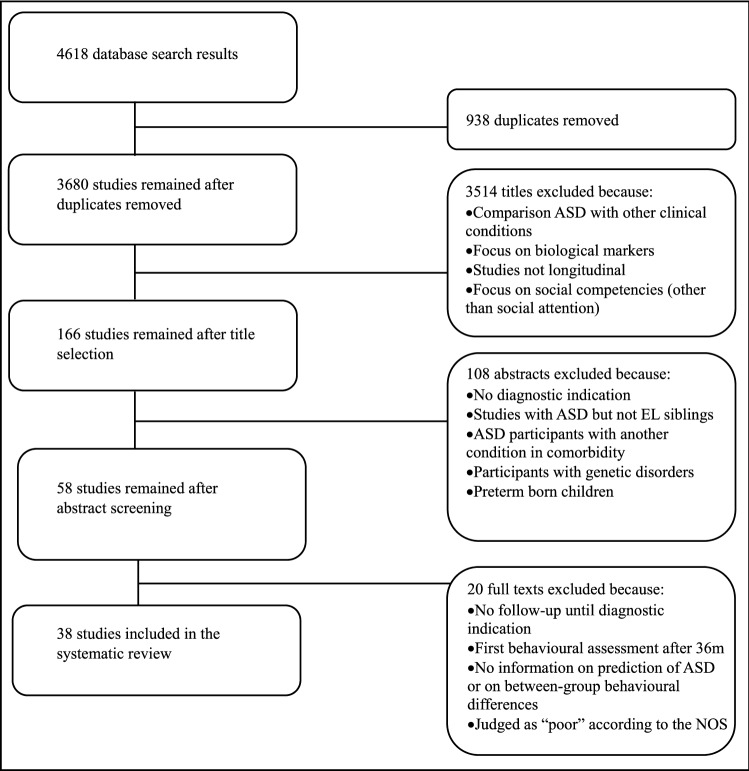
PRISMA flowchart of retrieved studies

The initial database search yielded 4618 articles. After removing the duplicates 3680 articles remained. In the following stages the two independent reviewers screened titles and abstracts for eligibility. After title screening 166 articles remained. In this phase, abstracts were inspected for all articles that appeared to be related to the subject. A total of 58 abstracts met the inclusion criteria. 38 articles were included in the systematic review after a close reading of the full texts. For a more schematic representation of the process, implemented according to the PRISMA guidelines, see Fig. 2.

## Results

Through the application of the NOS guidelines, 31 studies were scored as “good”, 7 studies as “fair”, 1 study as “poor”. This last study was excluded from further examination. Details on the evaluation of risk of bias for each study are reported in the Table in the supplementary material.

**Table 1:** Summary of the results

Domain	Sub-domain	Differences between EL-ASD and EL-TD	Age of appearance	Main papers reporting results
Attention	Disengagement of attention	Disengagement of attention predictor of ASD	12–13 months	Bedford et al. [31], Bryson et al. [32], Zwaigenbaum et al. [33]
	Attention engagement	No significant between-group difference	–	Sacrey et al. [19], Gammer et al. [29]
	Attention shift	No significant between-group difference	–	Bryson et al. [32], Zwaigenbaum et al. [33]
	Visual tracking	Inconclusive results:		
		No significant between-group difference versus	–	Gammer et al. [29]
		Visual tracking predictor of ASD	12 months	Zwaigenbaum et al. [33]
	Sustained attention	Inconclusive results:		
		Significant between-group difference versus	12 months	Zwaigenbaum et al. [33]
		No significant between-group difference	–	Wass et al. [34]
	Saccadic inhibitory control	Inconclusive results (1 study):		
		No significant between-group difference	–	Pijl et al. [35]
	Visual search	Inconclusive results:		
		Visual search predictor of ASD versus	15 months, 2 years	Gammer et al. [29]
		No significant between-group difference	–	Cheung et al. [36]
	Social Attention without social interaction	Inconclusive results:		
		1. Face processing: Predictor of ASD versus	14 months	de Klerk et al. [37]
		No significant between-group difference	–	Rutherford et al. [38]
		2. Gaze following behaviour: Significant between-group difference	13 months	Bedford et al. [39]
		3. Perception of biological motion: No significant between-group difference	–	Falck-Ytter et al. [40]
Visual processing	–	Inconclusive results:		
		Significant between-group difference versus	From 14 months on	Landa et al. [41]
		No significant between-group difference	–	Libertus et al. [42], Kaur et al. [43], Germani et al. [44]
Executive functioning	Working memory	Inconclusive results (1study):		
		No significant between-group difference	–	St. John et al. [45]
	Response inhibition	Inconclusive results (1study):		
		No significant between-group difference	–	St. John et al. [45]
Motor development	Motor control	Inconclusive results (1 study):		
		Motor control predictor of ASD	18 months	Brian et al. [46]
	General motor behaviour	Inconclusive results (1 study):		
		No significant between-group difference	–	Brian et al. [46]
	Gross motor development	Significant between-group difference	14 months	Landa et al. [41], Landa et al. [47]
	Fine motor development	Significant between-group difference	From 12 months on	Landa et al. [41], Landa et al. [47], Choi et al. [48]
	Posture	Inconclusive results (1 study):		
		No significant between-group difference	–	Nickel et al. [27]
Repetitive/stereotyped behaviour	Repetitive/restricted behaviour	Significant between-group difference	18 months	Sacrey et al. [49], Chawarska et al. [50]
	Repetitive body movement	Inconclusive results:		
		Significant between-group difference versus	12 months	Elison et al. [51]
		No significant between-group difference	–	Damiano et al. [52]
	Repetitive use of objects	No significant between-group difference	–	Elison et al. [51], Damiano et al. [52]
	Repetitive interests	Inconclusive results (1 study):		
		Significant between-group difference	6–12 months	Brian et al. [46]
Sensory Processing	Sensory behaviour and interests	Inconclusive results:		
		No significant between-group difference versus	–	Brian et al. [46]
		Sensory-oriented behaviour predictor of ASD	12 months	Zwaigenbaum et al. [33]
	Responses to sensory stimuli	Significant between-group difference (tactile domain)	From 6 or 12 months on	Sacrey et al. [19], Wolff et al. [53]
		Significant between-group difference (auditory domain)	24 months	Germani et al. [44], Wolff et al. [53]
Play	General play behaviour	Inconclusive results (1 study):		
		Significant between-group difference	9 months	Sacrey et al. [19]
	Functional play	Inconclusive results (1 study):		
		No significant between-group difference	–	Christensen et al. [54]
	Non-functional play	Inconclusive results (1 study):		
		No significant between-group difference	–	Christensen et al. [54]
	Symbolic play	Inconclusive results (1 study):		
		No significant between-group difference	–	Christensen et al. [54]
Temperament	Reactivity	Inconclusive results (1 study):		
		Significant between-group difference	18 months	Brian et al. [46]
	Transition	Inconclusive results (1 study):		
		Significant between-group difference	18 months	Brian et al. [46]
	Surgency	Inconclusive results (1 study):		
		Significant between-group difference	From 8 months on	Pijl et al. [35]
	Positive affect	Significant between-group difference	12 months	Zwaigenbaum et al. [33], Garon et al. [55]
	Approach	Inconclusive results:		
		Significant between-group difference versus	6 months or 24 months	Zwaigenbaum et al. [33], Brian et al. [46], Garon et al. [55]
		No significant between-group difference	–	Del Rosario et al. [56]
	Activity	Inconclusive results:		
		Significant between-group difference versus	6 months	Brian et al. [46]
		No significant between-group difference from 18 months	–	del Rosario et al. [56]
	Adaptability	Inconclusive results (1 study):		
		Significant between-group difference	From 6 months on	Brian et al. [46]
	Effortful control	Significant between-group difference	From 14 or 24 months on	Zwaigenbaum et al. [33], Pijl et al. [35], Garon et al. [57]
	Emotion regulation	Inconclusive results (1 study):		
		No significant between-group difference	–	Garon et al. [57]

Through data extraction, eight behavioural domains were identified within the 38 included studies: attention (*n* = 15), visual processing (*n* = 6), executive functioning (*n* = 1), motor functioning (*n* = 11), repetitive/stereotyped behaviour (*n* = 5), sensory processing (*n* = 5), play (*n* = 2) and temperament (*n* = 6). Ten out of 38 studies explored at least two behavioural domains, the remaining focused on a single domain. Results will be presented separately for each domain. A synthetic description of the experimental design, participants, instruments and results for every study can be found in Table 1 and, separately for each domain, in Tables 2, 3, 4, 5, 6, 7, 8 and 9. Summary outcome measures are between-group differences in the aforementioned dimensions (i.e. first EL versus TL, and next EL-ASD versus EL-TD and EL-Other), their predictive role towards later ASD diagnosis, and their developmental evolution over time. Findings on a non-social domain are accounted as consistent if concordant results are reported by at least two articles, from different research groups. Additionally, specifications on the assessment instrument of non-social behaviour are provided when describing both concordant and discordant findings.

**Table 2:** Attention

Study	Age at different assessments (in months)	Total number of participants	Number of EL siblings	Number of siblings with ASD outcome	Number of siblings with TD outcome	Number of siblings with another outcome ^a^	Topic	Assessment method	Diagnostic instruments	Difference EL-ASD vs EL-TD And/or predicts ASD diagnosis	Main findings
Bedford et al. (2012) UK [39]	7, 13, 24, 36	73	35	12	14	9	Social attention without social interaction	Tobii 1750 (50Hz) Gaze following task	Autism Diagnostic Observation Schedule-Generic (ADOS-G) Autism Diagnostic Interview-Revised (ADI-R), expert clinical judgment according to ICD-10	Yes	At 7 m there was no difference between EL and TL or between EL-ASD, EL-Other, EL-TD and TL in the proportion of first look to congruent versus incongruent stimuli and in looking time towards congruent stimuli At 13 m EL-ASD showed reduced looking time to congruent stimuli compared to EL-TD and to TL and in EL-Other compared to EL-TD and to TL; no difference in the first look to congruent versus incongruent stimuli between any group was found
Bedford et al. (2014) UK/Canada [31]	13, 36	88	40	16	24	–	Disengagement of attention	Ocular-motor behaviour videotaped during the gap-overlap task and coded by raters	ADOS-G, ADI-R, expert clinical judgment according to ICD-10	Yes	Increased saccadic reaction time at 13 m predicted ASD outcome at 36 m
Bryson et al. (2018) Canada [32]	6, 12, 36	136	83	16	67	–	Disengagement of attention	Ocular-motor behaviour videotaped during the gap-overlap task and coded by raters	ADOS-G, ADI-R, expert clinical judgment according to DSM-IV-TR	Yes	Disengagement of attention: at 6 m no between group difference was found at 12 m EL-ASD had longer disengage latencies (particularly for latency towards stimuli appearing on the left) than EL-TD and TL. Disengage latencies did not differ between EL-TD and TL only EL-ASD showed an increase in left-sided saccadic latency from 6 to 12 m Disengagement (right and left latencies) at 12 m predicted ADOS severity score at 36 m
							Attention shift				Attention shift: no group differences in the shift trials were found
Cheung et al. (2018) UK [36]	9, 15, 27, 36	140	113	17	64	32	Visual search	Tobii T120 visual search task	Autism Diagnostic Observation Schedule-2 (ADOS-2), ADI-R, expert clinical judgment according to DSM-5	Yes	At 9 m EL-ASD showed longer looking time and higher number of valid trials than EL-TD and TL, while no difference was found between EL-ASD and EL-Other. EL-ASD showed higher proportion of first look to the target than EL-Other and TL No between group differences at 15 m and 2 years in looking time and number of valid trials At 15 m but not at 2 years EL-ASD showed superior visual search performance than EL-Other and TL
Elsabbagh et al. (2011) UK/Canada [58]	10, 36–48	27	27	Not reported	Not reported	–	Saccadic inhibitory control	Freeze-frame task (videotaped and coded)	ADOS-G	Not reported	At 10 m EL who looked more at the boring central targets than at the distractors had a higher level of impairment in social skills at 36-48 m, compared to TL
Elsabbagh et al. (2013) UK [59]	6–10, 12–15, 24, 36	104	54	Not reported	Not reported	–	Disengagement of attention	Ocular-motor behaviour videotaped during the gap-overlap task and coded by raters	ADOS-G, ADI-R, expert clinical judgment according to ICD-10	Yes	During the first year no between-group difference in reaction time was found At 14 m EL-ASD had prolonged overlap RT compared to EL-TD, EL-Other and TL Different developmental courses were described: TL and EL-TD but not EL-ASD showed decreased RT in the overlap task over time
Elsabbagh et al. (2013) UK/Canada [60]	7, 14, 24, 36	103	53	17	24	12	Social attention without social interaction	Tobii (50 Hz) Face pop-out task	ADOS-G, ADI-R expert clinical judgment according to ICD-10	No	At 7 m and 14 m the proportion of trials with first look at faces was above chance in all groups At 14 m EL explored fewer AOIs than TL but this effect was not explained by the diagnosis EL spent more time than TL looking at the face area than other AOIs, and this effect was more clear at 14 m than 7 m
Falck-Ytter et al. (2018) Sweden/UK [40]	10, 36	47	33	13	12	−	Social attention without social interaction: multisensory integration during biological motion	Tobii 1750 & Tobii TX300 Point light animations	ADOS-G/ADOS-2, ADI-R, expert clinical judgment according to DSM-5	No	EL-ASD at 10 m were less able to differentiate between audiovisual synchrony, synchronous to the upright animation, versus synchronous to the inverted/reversed animation compared to EL-TD and TL No between-group difference was found in the preference for the upright compared to the inverted animation
Gammer et al. (2015) UK/Canada [29]	7, 14, 24, 36	104	54	17	36		Visual tracking	Autism Observation Scale for Infants (AOSI)	ADI-R, Social Communication Questionnaire (SCQ), ADOS-G, expert clinical judgment according to ICD-10	No	At 7 m EL-TD showed poorer visual tracking scores than TL
							Attention engagement				At 14 m between-group differences in engagement of attention were not significant after post-hoc tests
Gliga et al. (2015) UK [61]	9, 15, 24	109	82				Visual search	Tobii T120 visual search task	AOSI (15 m) ADOS-2 (24 m)	Yes	Visual search at 9 m predicted ASD symptoms at 15 m and ADOS score at 24 m
de Klerk et al. (2014) UK [37]	7, 36	84	44	14	19	11	Social attention without social interaction	Tobii (50 Hz) Face recognition task	ADOS-G, ADI-R, expert clinical judgment according to ICD-10	No	Face recognition task: at 7 m TL performed above chance level on easy and difficult items; EL only on easy items; there was no difference between each EL subgroup (EL-ASD, EL-TD, EL-Other) and TL, and no association between ASD-like characteristics and the performance on the task
								Face pop-out task			Face pop-out task: longer looking time at faces at 7 m was associated with poorer face recognition at 36 m in EL, but not in TL
Rutherford et al. (2015) US [38]	3, 6, 9, 12, 36	62	31	10	21		Social attention without social interaction	Tobii 60x (60 Hz) Face’s free viewing	ADOS-2	Yes	At 3 m EL-TD looked longer at the eye region than TL, EL-ASD looked at the eye region less than the other two groups At 3 m EL-TD had a bigger preference towards eyes over mouth than EL-ASD, while difference between EL-TD and TL and between EL-ASD and TL was not significant after Bonferroni correction At 6 m, 9 m, 12 m the between-group differences were not significant Across the first year EL-TD showed a decreased preference for the eyes, the same change was not significant for EL-ASD and was marginally significant for TL
Sacrey et al. (2013) Canada [62]	6, 9, 12, 15, 18, 24, 36	30	20	10	10		Disengagement of attention	AOSI, ADOS-G (videorecorded and coded)	ADOS-G, ADI-R, expert clinical judgment according to DSM-IV-TR	Yes	Disengagement of attention: EL-ASD were less likely to look away from the target before the grasp was complete and during the grasp compared to EL-TD and TL. Group differences started at 12 m, but were no longer evident at 36 m
							Attention engagement				Attention engagement: EL-TD and TL were more likely to move their hand towards a target before visually engaging it, compared to EL-ASD
							Sustained attention				Sustained attention: EL-ASD disengaged and re-engaged the target prior grasp more than TL
Wass et al. (2015) UK [34]	8, 36	94	45	15	30		Sustained attention	Tobii 1750 (50 Hz) Free viewing of static scenes	ADOS-G, ADI-R, expert clinical judgment according to ICD-10	Yes	At 8 m shorter fixation duration was found in EL than in TL and in EL-ASD than in TL; no significant difference was present between EL-TD and TL or between EL-ASD and EL-TD Shorter fixation duration at 8 m was associated with higher scores on the social communication ADOS scale at 36 m
Zwaigenbaum et al. (2005) Canada [33]	6, 12, 24	88	65	7	46	12	Disengagement of attention	Visual orienting task (videorecorded), AOSI	ADOS-G, ADI-R, expert clinical judgment according to DSM-IV	Yes	Disengagement of attention: between 6 and 12 m EL showed prolonged latencies in attention disengagement compared to TL while no difference was seen at 6 m. Disengagement score from the AOSI at 12 m predicted ASD at 24 m
							Attention shift and engagement				Attention engagement: EL-ASD at 12 m showed longer duration of orienting to objects than EL-TD and TL and less attention shifting at 24 m Attention shift: results showed no between-group difference in the latency to shift attention
							Sustained attention				Sustained attention: at 12 m EL-ASD had longer fixations on specific objects than EL-TD and TL

^a^Atypical outcome: deficit in general cognition, motor functioning, language delay, Broader Autism Phenotype (BAP, social communication delay)

**Table 3:** Visual processing

Study	Age at different assessments (in months)	Total number of participants	Number of EL siblings	Number of siblings with ASD outcome	Number of siblings with TD outcome	Number of siblings with another outcome^a^	Topic	Assessment method	Diagnostic instruments	Difference EL-ASD vs EL-TD and/or predicts ASD diagnosis	
Estes et al. (2015) US [26]	6, 12, 24	308	210	31	161	18	Visual processing	Mullen Scale of Early Learning (MSEL)	ADOS-G, ADI-R, expert clinical judgment according to DSM-IV-TR	No	On the Visual Reception scale: at 6 m EL-Autism (autism diagnosis) had a significantly lower score than TL no difference between EL-ASD, EL-TD and TL was found at 12 m EL-ASD showed a lower score than TL at 24 m EL-Autism had a lower score than EL-TD and TL EL-ASD had a lower score than TL
Germani et al. (2014) Canada [44]	24, 36	90	59	14	45		Visual processing	Infant Toddler Sensory Profile (ITSP)	ADOS-G, ADI-R, MSEL, expert clinical judgment according to DSM-IV-TR	No	At 24 m no difference was found between EL-ASD, EL-TD and TL
Kaur et al. (2015) US [43]	6, 9, 12, 15, 18, 24	32	16 (2 preterm)	3	8	5	Visual processing	Infants seated in a booster seat. Objects presented: a rattle, a koosh ball, a rigid ball	Ages and Stages Questionnaire-third edition (ASQ-3), Modified Checklist for Autism in Toddlers (M-CHAT), follow-up inquires with parents	No	EL showed a general trend of excessive visual exploration of objects, irrespective of the novelty of the objects (i.e. excessive looking at the rattle at 6 m and at the koosh ball at 12 m), compared to TL TL but not EL showed increased looking at the koosh ball from 12 to 15 m
Landa et al. (2006) US [47]	6, 14, 24	87	87	24	52	11	Visual processing	MSEL	ADOS, expert clinical judgment according to DSM-IV	Yes	At 14 m EL-ASD showed the same score in visual processing as EL-TD EL-ASD showed a lower increase over time than EL-TD EL-ASD showed the lowest increase over time
Landa et al. (2012) US [41]	6, 14, 18, 24, 30, 36	204	204	52	121	31	Visual processing	MSEL	ADOS-G, expert clinical judgment	Yes	EL-ASD were more likely assigned to the developmental slowing class compared to EL-TD (typical functioning at 6 m followed by attenuation in developmental rate and severe delay in visual processing) EL-Other (BAP) were assigned to normative class (normative visual processing development)
Libertus et al. (2014) US [42]	6, 36	129	107	22	57	28	Visual processing	MSEL	ADOS-G, expert clinical judgment according to DSM-IV	No	At 6 m no effect of group on the Visual Reception scale was found

^a^Atypical outcome: deficit in general cognition, motor functioning, language delay, Broader Autism Phenotype (BAP, social communication delay)

**Table 4:** Executive functioning

Study	Age at different assessments (in months)	Total number of participants	Number of EL siblings	Number of siblings with ASD outcome	Number of siblings with TD outcome	Number of siblings with another outcome^a^	Topic	Assessment method	Diagnostic instruments	Difference EL-ASD vs EL-TD and/or predicts ASD diagnosis	Main findings
St. John et al. (2018) US [45]	12, 24	262	186	19	106	–	Executing functioning: working memory (WM), response inhibition	A-not-B task	ADOS-2, ADI-R, expert clinical judgment according to DSM-IV-TR	No	At 12 m no between-group difference in WM or response inhibition was found From 12 to 24 m the performance of TL, but not of EL-ASD and EL-TD improved At 24 m EL-ASD and EL-TD showed worse WM and inhibition performance compared to TL. No significant difference between EL-ASD and EL-TD was found

^a^Atypical outcome: deficit in general cognition, motor functioning, language delay, Broader Autism Phenotype (BAP, social communication delay)

**Table 5:** Motor functioning

**Study**	Age at different assessments (in months)	Total number of participants	Number of EL siblings	Number of siblings with ASD outcome	Number of siblings with TD outcome	Number of siblings with another outcome^a^	Topic	Assessment method	Diagnostic instruments	Difference EL-ASD vs EL-TD and/or predicts ASD diagnosis	Main findings
Brian et al. (2008) Canada [46]	6–12, 18, 36	228	155	35	120		Motor control, general motor behaviour	ADOS-G, AOSI	ADOS-G, ADI-R, expert clinical judgment according to DSM-IV-TR	Yes	At time 1 (6-12 m): no motor impairment was identified in any participant At time 2 (18 m): EL-ASD showed atypical motor behaviour compared to EL-TD and to TL and abnormal motor control compared to TL Motor control predicted, with other dimensions, ASD at 36 m
Choi et al. (2018) US [48]	6, 12, 18, 24, 36	170	101	30	71	-	Fine motor milestones	MSEL	ADOS-G	Yes	At 12 m no difference between EL-ASD, EL-TD and TL EL-ASD had lower fine motor skills than EL-TD at 12 m and than TL at 18 m EL-ASD had lower growth rate than TL but not compared to EL-TD from 6 to 24 m
Estes et al. (2015) US [26]	6, 12, 24	308	210	31	161	18	Fine and gross motor milestones	MSEL	ADOS, ADI-R, expert clinical judgment	No	At 6 m EL-Autism had lower gross motor scores than TL At 24 m EL-Autism had lower scores in both Gross and Fine motor scales than EL-TD and TL. EL-ASD had lower scores in Fine motor scale than TL
Kaur et al. (2015) US [43]	6, 9, 12, 15, 18, 24	32	16 (2 preterm)	3 (2 preterm)	8	5	Fine motor milestones (grasping, dropping, mouthing)	Infants seated in a booster seat. Objects presented: a rattle, a koosh ball, a rigid ball	ASQ-3, M-CHAT, follow-up inquiries with parents	No	Grasping: EL showed less grasping of the rigid ball at 6 m than TL. Between 6 and 9 m TL had reduced grasping of the rigid ball; EL had increased grasping of the rigid ball and rattle. Between 9 and 12 m TL increased grasping of the rattle and EL increased grasping of the koosh ball Dropping: EL at 6 m showed much less dropping of the rigid ball, more dropping of objects from 6 to 9 m but lower level of dropping from 12 to 15 m compared to TL. TL showed increased dropping of objects from 6 to 9 m, while EL had delayed increase in dropping from 12 to 15 m Mouthing: EL showed less mouthing of the rattle at 6 m and more mouthing of the rattle and rigid ball at 15 m compared to TL
Iverson et al. (2019) US [63]	6, 36	625	437	69	317	51	Fine and gross motor milestones	MSEL	ADOS, expert clinical judgment according to DSM-IV-TR	Yes	Fine motor but not gross motor scores at 6 m predicted ASD at 36 m
Landa et al. (2006) US [47]	6, 14, 24	87	87	24	52	11	Fine and gross motor milestones	MSEL	ADOS-G, expert clinical judgment according to DSM-IV	Yes	At 14 m EL-ASD had a lower score than EL-TD in Gross motor and Fine motor scales EL-ASD showed a lower increase over time than EL-TD
Landa et al. (2012) US [41]	6, 14, 18, 24, 30, 36	204	204	52	121	31	Fine and gross motor milestones	MSEL	ADOS-G, expert clinical judgment	Yes	EL-ASD were more likely assigned to the developmental slowing class (typical functioning at 6 m followed by attenuation in developmental rate and severe delay in fine and gross motor development) compared to EL-TD EL-Other (BAP) were assigned to normative class or language/motor delay class (fine motor delay at 6 m followed by normative development in all areas except in gross and fine motor development)
LeBarton et al. (2019) US [64]	6, 24, 30, 36	140	89	20	69	–	Fine (visual-motor integration, grasping) and gross (stationary) motor milestones	Peabody Developmental Motor Scales – 2 (PDMS2)	ADOS-G	Yes	At 6 m EL-ASD showed worse visual-motor integration than TL; no significant between-group difference at 6 m in stationary and grasping was found Visual-motor integration at 6 m predicted ASD at 24–36 m
Libertus et al. (2014) US [42]	6, 36	129	107	22	57	28	Fine and gross motor milestones	MSEL	ADOS-G, expert clinical judgment according to DSM-IV	No	At 6 m: for gross motor development, no between-group difference was found for fine motor development, significantly higher scores in TL than EL-TD, EL-Other and EL-ASD were found. Post-hoc comparison showed no differences between the 3 EL subgroups
Nickel et al. (2013) US [27]	6, 9, 12, 14, 18, 21, 24, 36	40	22	4 (EL-Autism)	18	–	Posture	Home videos videotaped and coded	ADOS-G, expert clinical judgment according to DSM-IV	Yes	From 6 to 12 m EL-Autism were seen in half as many different postures as EL-TD and TL (positions at 6 m: prone, supine, sit supported, sit unsupported, all-4) but by 14 m differences were no longer visible
Sacrey et al. (2018) Canada [49]	6, 9, 12, 15, 18, 24, 3	30	10	10	10	–	Fine and gross motor milestones: reach-to-grasp movement (orient, lift, pronate, grasp)	Videos during AOSI, ADOS-G, videotaped and scored using the Skilled Reaching Rating Scale	ADOS-G, ADI-R, expert clinical blind assessment according to DSM-IV-TR	Yes	EL-ASD had worse reach-to-grasp scores than EL-TD and TL (which did not differ); no group × age interaction was found EL-ASD showed: worse orient and lift than TL but did not differ from EL-TD worse pronate scores than EL-TD and TL no difference in grasp from any other group Improvement in orienting started between 9 and 12 m and in lifting and pronate from 6 m

^a^Atypical outcome: deficit in general cognition, motor functioning, language delay, Broader Autism Phenotype (BAP, social communication delay)

**Table 6:** Repetitive/stereotyped behaviour

Study	Age at different assessments (in months)	Total number of participants	Number of EL siblings	Number of siblings with ASD outcome	Number of siblings with TD outcome	Number of siblings with another outcome^a^	Topic	Assessment method	Diagnostic instruments	Difference EL-ASD vs EL-TD and/or predicts ASD diagnosis	Main findings
Brian et al. (2008) Canada [46]	6–12, 18, 36	228	155	35	120	–	Repetitive/stereotyped behaviour; repetitive interests	ADOS-G, AOSI	ADOS-G, ADI-R, expert clinical judgment according to DSM-IV-TR	Yes	At 6-12 m EL-ASD had more repetitive interests than EL-TD and TL; EL-TD showed significantly more repetitive interests than TL
Chawarska et al. (2014) US/Canada [50]	18, 36	719	719	157	384	178	Repetitive/stereotyped behaviour	ADOS-G, MSEL	ADOS-G, expert clinical judgment according to DSM-5	Yes	In a subgroup of EL-ASD repetitive behaviour at 18 m predicted ASD outcome
Damiano et al. (2013) US [52]	12–24, 24–36	40	20	8	12	–	Repetitive/stereotyped behaviour: repetitive body movements and use of objects	Repetitive and Stereotyped Movement Scales (RSMs)	ADOS-G, ADI-R, expert clinical judgment	No	At 12–24 m EL had higher rates of RSMs than TL At 24–36 m EL kept higher rates of RSMs than TL, even when analyses were repeated excluding EL-ASD subgroup In EL-TD but not in EL-ASD the object RSM inventory score was higher than the body RSM inventory score
Elison et al. (2014) US/Canada [51]	12, 24		105	30	75	–	Repetitive/stereotyped behaviour: stereotyped behaviour, repetitive object manipulation, repetitive body movements	RSMs, MSEL	ADOS-G, expert clinical judgment according to DSM-IV	Yes	On the object cluster subscale at 12 m: EL-ASD and EL-TD scored significantly higher than TL no significant difference was found between EL-ASD and EL-TD On the body cluster subscale at 12 m: EL-ASD scored significantly higher than EL-TD and TL no significant difference between EL-TD and TL was found
Sacrey et al. (2015) Canada [19]	6, 9, 12, 15, 18, 24, 36–42	237	168	62	106	–	Repetitive/stereotyped behaviour	MSEL, The Vineland Adaptive Behaviours Scales (VABS), parent concerns interview	ADOS-G, ADI-R, expert clinical judgment according to DSM-IV	Yes	Parents of EL-ASD had more concerns about repetitive and restricted behaviour compared to parents of TL since 9 m and compared to parents of EL-TD since 18 m

^a^Atypical outcome: deficit in general cognition, motor functioning, language delay, Broader Autism Phenotype (BAP, social communication delay)

**Table 7:** Sensory processing

Study	Age at different assessments (in months)	Total number of participants	Number of EL siblings	Number of siblings with ASD outcome	Number of siblings with TD outcome	Number of siblings with another outcome^a^	Topic	Assessment method	Diagnostic instruments	Difference EL-ASD vs EL-TD and/or predicts ASD diagnosis	Main findings
Brian et al. (2008) Canada [46]	6–12, 18, 36	228	155	35	120	–	Sensory processing: sensory behaviour and interests	ADOS-G, AOSI	ADOS-G, ADI-R, expert clinical judgment according to DSM-IV-TR	No	On the subscale for atypical sensory behaviour at 18 m: EL-ASD showed a higher score than TL, while differences between EL-ASD and EL-TD were no longer present after Bonferroni correction EL-TD showed a higher score than TL
Germani et al. (2014) Canada [44]	24, 36	90	59	14	45	–	Sensory processing: auditory, tactile, vestibular, oral domains	Infant Toddler Sensory Profile (ITSP)	ADOS-G, ADI-R, MSEL, expert clinical judgment according to DSM-IV-TR	Yes	EL-ASD at 24 m scored significantly higher than EL-TD and TL in items for auditory processing; EL-TD and TL did not differ significantly No difference for visual, tactile, vestibular, oral domains was found
Sacrey et al. (2015) Canada [19]	6, 9, 12, 15, 18, 24, 36–42	237	168	62	106	–	Sensory processing: general	MSEL, VABS, parents‛ interview	ADOS-G, ADI-R, expert clinical judgment according to DSM-IV	Yes	Parents of EL-ASD had more sensory concerns by 6 m and 9 m than parents of EL-TD and TL (who did not differ)
Wolff et al. (2018) US/Canada [53]	12, 14	466	331	74	257	–	Sensory responsivity, sensory interests: auditory, tactile, visual domains	Sensory Experiences Questionnaire (SEQ)	ADOS-G, ADIR-R, MSEL, expert clinical judgment according to DSM-IV-TR	Yes	At 12 m EL-ASD showed higher scores in sensory hyper-responsivity than EL-TD and TL and in the tactile modality than EL-TD From 12 to 24 m SEQ total score, hypo-responsivity and visual modality scores increased for EL-ASD and decreased for EL-TD At 24 m EL-ASD had higher scores in all subtests than EL-TD
Zwaigenbaum et al. (2005) Canada [33]	6, 12, 24	88	65	7	46	12	Sensory processing: sensory behaviour	AOSI	ADOS-G, ADI-R, expert clinical judgment according to DSM-IV	Yes	At 12 m but not at 6 m atypical sensory-oriented behaviour predicted ASD at 24 m

^a^Atypical outcome: deficit in general cognition, motor functioning, language delay, Broader Autism Phenotype (BAP, social communication delay)

**Table 8:** Play

Study	Age at different assessments (in months)	Total number of participants	Number of EL siblings	Number of siblings with ASD outcome	Number of siblings with TD outcome	Number of siblings with another outcome^a^	Topic	Assessment method	Diagnostic instruments	Difference EL-ASD vs EL-TD and/or predicts ASD diagnosis	Main findings
Christensen et al. (2010) US [54]	6, 12, 18, 36	77	58	17	29	12	Play: functional repeated play non-functional repeated play, symbolic play	Free play-assessment (based on set of toys) videotaped and coded	MSEL,VABS, parent concerns’ interview	No	At 18 m: EL-ASD had fewer novel other-directed functional play than TL EL-ASD showed greater levels of non-functional repeated play than TL (but the effect dropped out when controlling for verbal age); EL-TD showed more non-functional repeated play than TL no between-group difference in symbolic play no between-group difference in functional repeated play no difference between EL-Other, EL-TD and TL on novel functional play
Sacrey et al. (2015) Canada [19]	6, 9, 12, 15, 18, 24, 36–42	237	168	62	106	–	Play: parents’ concerns	Parent concerns’ interview	ADOS-G, ADI-R, expert clinical judgment according to DSM-IV	Yes	At 9 m: EL-ASD showed poorer play skills than EL-TD and TL according to parents’ concerns EL-TD and TL did not differ after post-hoc analysis

^a^Atypical outcome: deficit in general cognition, motor functioning, language delay, Broader Autism Phenotype (BAP, social communication delay)

**Table 9:** Temperament

Study	Age at different assessments (in months)	Total number of participants	Number of EL siblings	Number of siblings with ASD outcome	Number of siblings with TD outcome	Number of siblings with another outcome^a^	Topic	Assessment method	Diagnostic instruments	Difference EL-ASD vs EL-TD and/or predicts ASD diagnosis	Main findings
Brian et al. (2008) Canada [46]	6–12 (time 1), 18 (time 2), 36	228	155	35	120	–	Temperament: transition, reactivity	ADOS-G, AOSI	ADOS-G, ADI-R, expert clinical judgment according to DSM-IV-TR	Yes	At time 2 (18 m): EL-ASD showed higher scores on transition compared to EL-TD and to TL EL-ASD showed higher levels of reactivity compared to EL-TD and TL transition and reactivity predicted ASD at 36 m
del Rosario et al. (2014) US [56]	6, 12, 18, 24, 36	37	37	27	10	–	Temperament: Activity, Adaptability, Approach	Carey Temperament Scale (CTS) in 3 versions: Revised Infant Temperament Questionnaire (RITQ) at 6 m, Toddler Temperament Questionnaire (TTQ) at 12 m and 24 m, Behaviour Style Questionnaire (BSQ) at 36 m	ADOS-G, SCQ, expert clinical judgment according to DSM-IV-TR	Yes	On adaptability scale, EL-ASD had lower score than EL-TD at 6 m and at 12 m; EL-ASD had higher score than EL-TD at 24 m and 36 m On the approach scale, EL-ASD had lower score than EL-TD at 6 m; EL-ASD had higher score than EL-TD at 24 m and 36 m EL-ASD showed less active behaviour than EL-TD at 6 m and at 12 m but no differences afterwards
Garon et al. (2009) Canada [57]	24, 36	192	138	34	104	Not reported	Temperament: behavioural approach, effortful emotion regulation	Toddler Behaviour Assessment Questionnaire-Revised (TBAQ-R)	ADOS-G, ADI-R, MSEL, expert clinical judgment according to DSM-IV-TR	Yes	At 24 m: EL-ASD scored significantly lower on behavioural approach than EL-TD and TL while EL-TD scored higher than TL TL had significant higher score on emotion regulation than EL-ASD and EL-TD, while EL-ASD and EL-TD did not show a significant difference 65% of EL-ASD were below average on behavioural approach and effortful emotion regulation; 74% of EL-TD showed higher than average behavioural approach and lower effortful emotion regulation; 70% of TL had higher than average effortful emotion regulation Behavioural approach better discriminated between EL-ASD and EL-TD than effortful emotion regulation. Effortful emotion regulation better discriminated between EL-ASD and TL than behavioural approach, although both were significant. Both functions distinguished EL-TD and TL, with behavioural approach being slightly higher than effortful emotion regulation
Garon et al. (2016) Canada [55]	6–12, 24, 36	534	373	29	278	Not reported	Temperament: positive affect, effortful control	Infant Behavior Questionnaire (IBQ) at 12 m, TBAQ-R at 24 m	ADOS-G, ADI-R, MSEL, expert clinical judgment according to DSM-IV-TR	Yes	At 12 m EL were rated as higher on distress to limitations and fear compared to TL At 24 m EL were rated as higher on fear, sadness, anger, and lower on inhibitory control, soothability, attention focus, high pleasure, low pleasure than TL For EL, positive affect at 12 m predicted ASD symptoms at 36 m. This relationship was indirect and mediated by effortful control at 24 m. Lower effortful control score at 24 m predict more ASD symptoms at 36 m
Pijl et al. (2019) UK/NL [35]	8, 14, 24, 36	199	133	24	75	34	Temperament: surgency, negative affect, effortful control	IBQ-R at 8 m and 14 m, Early Childhood Behavior Questionnaire (ECBQ) at 24 m	ADOS-2, ADI-R, expert clinical judgment according to DSM-5	Yes	From 8 to 14 m EL-ASD had lower surgency scores than EL-TD and TL EL-ASD from 8 m on showed higher negative affect than EL-TD, EL-Other and TL EL-ASD at 14 m and at 24 m showed lower effortful control than EL-Other, EL-TD and TL A combination of all above temperament dimensions at 24 m as well as effortful control at 14 m and effortful control + negative affect at 24 m predicted ASD
Zwaigenbaum et al. (2005) Canada [33]	6, 12, 24	88	65	7	46	12	Temperament: inhibitory control, activity level, distress reactions, positive anticipation, affective responses	IBQ (at 6, 12 m), TBAQ (at 24 m)	ADOS-G, ADI-R, expert clinical judgment according to DSM-IV	Yes	Parents of EL-ASD described their children as having lower activity level at 6 m and more frequent and intense distress reactions, less inhibitory control, less positive anticipation and affective responses at 12 m compared to EL-TD and TL children

^a^Atypical outcome: deficit in general cognition, motor functioning, language delay, Broader Autism Phenotype (BAP, social communication delay)

### Attention (*n* = 15; Table 2)

The vast number of studies covered seven aspects or subcomponents of attention development: disengagement of attention, attention shift and engagement, visual tracking, sustained attention, saccadic inhibitory control, visual search, and social attention without social interaction.

#### Disengagement of attention (*n* = 5)

Attention disengagement was explored through a visual orienting task in three studies [31, 32, 59] various play situations in one study [62], and using both a visual orienting task and a play situation in one study [33]. In the visual orienting task, a stimulus is presented on the right or left of the screen while the participant is looking at a central stimulus. Three trial types can alternate: the overlap condition in which the central fixation and the peripheral stimulus are extinguished simultaneously, the gap condition, in which the central fixation extinguishes before the peripheral stimulus appears and the baseline condition in which the central fixation stimulus extinguishes as soon as the peripheral stimulus appears. Two out of four studies [31, 59] included overlap and baseline conditions, the remaining [32, 33] considered overlap and gap conditions. Attention disengagement was conceptualised, across studies, as the latency to make an eye movement towards a peripheral stimulus while the subject is engaged on a central fixation stimulus [31, 32, 59]. In Bedford et al. [31] and Elsabbagh et al. [59], disengagement was calculated as the difference between the saccadic reaction time in overlap trials compared to baseline trials. Zwaigenbaum et al. [33] additionally explored the construct during a play situation from the Autism Observation Scale for Infants (AOSI; [65]), while Sacrey et al. [62] analysed play situations from both the AOSI and the Autism Diagnostic Observation Schedule (ADOS; [66]). The AOSI [65] is an observational measure based on a standard set of semi-structured activities, developed to detect early signs of autism as they emerge in EL infants. Attention disengagement is explored by shaking a rattle on one side of the infant while his attention is engaged on another rattle that has been previously presented on his other side. The ADOS [66] uses standardized activities to elicit communication, social interaction, imaginative use of play materials and repetitive behaviours. It assesses disengagement via looking at the time period between the target’s grasp and an eye movement away from the target. Results from the visual orienting task revealed that disengagement discriminated EL from TL between 6 and 12 months [33]. From the end of children’s first year and during the second year, differences were visible within the EL group. In particular, at 12 months [32] or at 14 months [59] EL-ASD required more time to disengage from a stimulus than EL-TD, EL-Other and TL during the overlap condition. Additionally, saccadic reaction times at 12–13 months predicted social-communicative impairments at 24 months [33], ASD outcome [31] or the severity of autistic symptoms [32], measured by the ADOS at 36 months.

Finally, different developmental courses were found: TL and EL-TD but not EL-ASD showed decreased RT over time (range 6–36 months) in the overlap condition [32, 59]. Similar results were found during play situations. Attention disengagement, measured through the AOSI [65] at 12 months, predicted ASD at 24 months, according to Zwaigenbaum et al. [33]. Sacrey et al. [62] reported that EL-ASD did not disengage from the target after it was grasped, as compared to EL-TD and TL, although this pattern was evident from 12 until 24 months, no longer at 36 months.

#### Attention shift and engagement (*n* = 4)

Sacrey et al. [62] and Gammer et al. [29] explored attention engagement during various play situations, as part of the AOSI [65] and ADOS [66]. In this context, they referred to engagement as the time period between the first eye movement towards the toy and the first hand movement towards that particular toy. Neither Sacrey et al. [62] nor Gammer et al. [29] found significant between-group differences in looking time towards the target before hand movement. Additionally, although EL-ASD were less likely than EL-TD and TL to look away from the target before the grasp was complete, these eye movements accounted for a small minority of all visual engagements while the majority of visual engagements toward the target were appropriate [62].

Explored using the gap condition from the visual orienting task [33] and a play situation from the AOSI [65], attention shift (rather than engagement) was defined by two studies [32, 33] as the latency to gaze to the peripheral stimulus after a central stimulus has disappeared. In line with the two previous studies, Zwaigenbaum et al. [33] and Bryson et al. [32] did not find between-group nor within-group differences in attention shift at any age; consistently, attention shift did not predict the scores on the ADOS [62] at 24 [33] or 36 months [32].

#### Visual tracking (*n* = 2)

Visual tracking was explored during the administration of the AOSI [65] in which it is defined as the ability to visually follow a moving object laterally across the midline. Gammer et al. [29] found that at 7 months but not at 14 months EL-TD showed higher scores in the visual tracking behaviour as compared to TL, although the authors did not report whether the scores indicate partial or full inability to track objects laterally. Conversely, EL-ASD and EL-TD did not significantly differ at any time point. Differently, Zwaigenbaum et al. [33] found that visual tracking at 12 months but not at 6 months predicted the ADOS score at 24 months.

#### Sustained attention (*n* = 3)

Sustained attention was measured during a play situation as part of the AOSI and ADOS [62], during the exploration of static scenes [34] and through the Infant Behavior Questionnaire (IBQ; [67]), a parent report about children’s exploration strategies in daily life [33]. Sacrey et al. [62] defined sustained attention as the continual visual engagement of the target from movement onset until the target has been grasped. Wass et al. [34] and Zwaigenbaum et al. [33] focused on the duration of fixations towards a stimulus, during a free play condition and through parent reports.

Results disclosed different patterns. Zwaigenbaum et al. [33] found that parents of EL-ASD described their children at 12 months with a tendency to fixate more on particular objects in the environment at the expense of a more active visual exploration, compared to parents of EL-TD and TL. Sacrey et al. [62] found that EL-ASD disengaged and engaged the target several times before grasping it, compared to EL-TD and TL, although the group by age interaction was no longer significant after post-hoc analyses. Wass et al. [34] found shorter fixation duration at 8 months in EL-ASD compared to TL, while EL-TD did not significantly differ from EL-ASD or TL.

#### Saccadic inhibitory control (*n* = 1)

Defined as the combination of attentional flexibility and regulation of looking behaviour in response to changes in visual environment, saccadic inhibitory control was assessed in one study [58] using the Freeze-frame Task [68]. Children’s task was to inhibit looking to peripherally presented distractors so as to keep a central stimulus animated. The authors measured the proportion of looks at distractors in boring and interesting trials and found that EL who looked more at central boring targets than at distractors at 10 months exhibited higher levels of impairment in social skills from 36 to 48 months compared to TL. No comparison within the EL group according to the diagnostic outcome was made.

#### Visual search (*n* = 2)

A visual search task, comprised of an array of coloured distractor-letters and one target-letter, disposed on an imaginary circle, was presented to EL and TL while their ocular-motor behaviour was recorded, in two studies [36, 61]. Gliga et al. [61] and Cheung et al. [36] operationalized visual search accuracy as the proportion of trials in which the participant made a first look toward the target. Both studies showed enhanced visual search performance in EL-ASD but while in Gliga et al. [61] visual search accuracy at 9 months predicted ASD symptoms already at 15 months and again at 2 years, Cheung et al. [36] concluded that between-group differences were no longer found at 2 years.

#### Social attention without social interaction (*n* = 5)

Overall, three components of social attention were explored: face processing, gaze following behaviour, orienting to biological motion. Face processing was conceived as a combination of face recognition, scanning and face preference [37, 38, 60]. Elsabbagh et al. [60] and de Klerk et al. [37] explored face processing through the face pop-out task. In the task, sequences of five images (one face and four distractors) appeared on the screen while participants’ eye movements were recorded. Distractors were cars, mobile phones, birds and face visual noise images from the same face presented in the sequence. The dependent variables were the proportion of trials with the first look at the face and the total amount of time spent looking at the face compared to the other areas of interest (AOIs). Results showed that EL looked at faces for a longer time than TL, and this pattern was clearer at 14 months than at 7 months. However, this finding was explained by the ASD diagnosis only in de Klerk et al. [37].

Rutherford et al. [38] explored face processing during the free viewing of various coloured dynamic videos each displaying a different face, several moments across development. They found that at 3 months EL-ASD showed the smallest preference for the eye region, followed by TL, who looked at the eye region less than EL-TD. Moreover, EL-TD showed a bigger preference for the eyes over the mouth than EL-ASD, while both EL-ASD versus TL and EL-TD versus TL did not differ after Bonferroni’s correction. In addition, between-group differences were no longer apparent at 6, 9 and 12 months. Finally, in EL-TD, and unlike EL-ASD, a decreased preference for eyes across the first year was found.

Gaze following behaviour was defined as the proportion of first looks to a congruent versus an incongruent object [39]. The visual stimuli consisted of two objects on a table and a female model looking down, then looking up (direct gaze) and then turning her head to look at one of the objects (shift). The object looked at by the model during shift was the congruent object and the non-gazed at object was the incongruent object. Bedford et al. [39] found that in the assessment at 13 months, but not at 7 months, EL-ASD looked at the congruent stimuli for less time than EL-TD and TL, while no difference was found between EL-ASD and EL-Other.

Lastly, perception of biological motion was conceptualized as an example of social information that is prioritized by typically developing infants and children [40]. Falck-Ytter et al. [40] assessed the effects of manipulation of audiovisual synchrony by presenting point light animations of diverse actions together with audio, with the same action being always shown in upright on one side of the screen and spatially inverted (and temporally reversed) on the other side. Sometimes the audio was coincidentally in synchrony with the inverted and reverse animation.

The authors found that EL-ASD oriented less to audiovisual synchrony expressed within biological motion, compared to EL-TD and TL. However, all groups showed a preference for the upright animation, suggesting that results are not due to differences in orienting to biological motion.

### Visual processing (*n* = 6; Table 3)

Visual processing was explored in three different manners: by the Visual Reception scale from the Mullen Scale of Early Learning (MSEL; [69]) in four studies [26, 41, 42, 47], in which a variety of abilities (i.e. visual discrimination, visual memory, visual organization, visual sequencing and visual-spatial awareness) were covered, by the overall looking behaviour during a free play situation, in one study [43], in which participants were presented with various objects of different size, shape, and texture, requiring different types of exploratory behaviours, and as one of the sensory domains of the parent questionnaire Infant Toddler Sensory Profile (ITSP; [70]), in one study [44].

Put together, results showed that abnormal visual processing at 6 months discriminated EL-Autism (referring to children with high ADOS scores, eligible for the diagnosis of Autism at 36 months) from EL-ASD, EL-TD and TL [41]. Differences were not disclosed before 14 [41] and 24 months [47] in EL-ASD compared to EL-TD and TL. Moreover, Landa et al. [41, 47] found a developmental trend characterised by a decrease in visual processing abilities between the first and the second birthday (and until 36 months—evaluation in Landa et al. [41]), although these findings came from the same research group. In contrast, Libertus et al. [42] did not find any difference at 6 months between EL-ASD and EL-TD but they did not include any other assessment between 6 and 36 months.

Kaur et al. [43] explored the overall looking behaviour towards a set of toys and found that EL showed excessive visual exploration irrespective of the novelty of the objects used compared to TL, at 6 months and 12 months while no difference was reported within the EL group. Germani et al. [44] conceptualized visual processing as one of the sensory processing subdomains and, in contrast with previously mentioned studies, did not find any significant difference at 24 months between EL-ASD, EL-TD and TL.

### Executive functioning (*n* = 1; Table 4)

St John et al. [45] assessed children’s executive functioning through the A-not-B task [71]. Participants needed to look as a toy was hidden to the right or left of the midline and were encouraged to find the toy after a few seconds delay. The side was reversed after the toy was found on two consecutive trials. Authors measured the proportion of total correct reaches by total trials, as an indicator of working memory (WM), and the proportion of total correct reaches on reversal trials by total reversals trials, which offers a measure of response inhibition.

Results showed an improvement in the WM and inhibition performance of TL between 12 and 24 months, compared to EL-ASD and EL-TD, who did not differ. No between-group differences in WM or response inhibition were found at 12 months. On the contrary, the WM and inhibition performance of EL-ASD and EL-TD at 24 months was significantly worse compared to TL, although no difference was found between the EL subgroups.

### Motor functioning (*n* = 11; Table 5)

Three sub-domains were identified: motor control and general motor behaviour, fine and gross motor milestones and posture.

#### Motor control and general motor behaviour (*n* = 1)

Motor control and general motor behaviour have been assessed by Brian et al. [46] during the administration of the AOSI [65]. In the AOSI motor control is defined as the degree in which motor behaviour is goal-directed, organised and modulated while general motor behaviour comprises atypical gait, locomotion, motor mannerism/postures or repetitive motor behaviour. The authors found that at 18 months motor behaviour was more atypical in EL-ASD compared to EL-TD and TL. Moreover, EL-ASD showed abnormal motor control compared to TL, while differences between EL-ASD and EL-TD were not significant after Bonferroni correction. Nonetheless, motor control at 18 months contributed to predict ASD at 36 months.

#### Fine and gross motor milestones (*n* = 9)

Motor milestones were assessed using four different measures: the MSEL [69], the Peabody Developmental Motor Scales-2 (PMDS-2; [72]), recordings from the AOSI [65] and ADOS [66] and a free play situation. From the MSEL the Gross motor scale, that assesses central motor control and mobility, and the Fine motor scale, an indicator of visual-motor ability, were considered [26, 41, 42, 47, 48, 63]. LeBarton et al. [64] used the PMDS-2 [72] a standardized, experimenter-administered observational measure, comprised of the Stationary subscale to test gross motor skills and the Grasping and Visual-Motor Integration subscales to test fine motor skills. Sacrey et al. [49] analysed the recordings from the AOSI [65] and ADOS [66] and coded the reach-to-grasp measures (which included orient, lift, advance, pronation and grasp). Finally, Kaur et al. [43] used a less structured play situation, in which various objects of different size, shape and texture were presented one at a time and grasping, dropping and mouthing were coded.

The pattern of gross motor development was coherent in most of the studies showing no difference in the gross motor abilities at 6 months between EL-ASD and EL-TD [41, 42, 47, 64]. Differences between EL-ASD and EL-TD appeared at 14 months according to two studies [41, 47]. Coherently, gross motor abilities at 6 months could not predict ASD at 36 months [63]. Differently, Sacrey et al. [49] identified worse scores in reach-to-grasp movements, particularly in orient (head and eye movements to fixate the target prior reaching it) and pronate (hand pronates over the target and digits shape to target size) movements in EL-ASD than EL-TD and TL from 6 months, although no Group × Age interaction was found.

Uniquely, Estes et al. [26] followed up EL children with a later diagnosis of Autism Disorder (EL-Autism) and found that they had a lower score on the Gross motor scale already at 6 months.

Results for the fine motor domain based on the MSEL [69] and the PMDS-2 [72] revealed that at 6 months fine motor competencies predicted 36 months ASD diagnosis, as reported by Iverson et al. [63], discriminated EL and TL according to Libertus et al. [42] and LeBarton et al. [64] but not according to Choi et al. [48]. Differences within the EL group between EL-ASD and EL-TD appeared during the second year (from 12 months in Choi et al. [48], at 14 months in Landa et al. [41, 47]) while in Estes et al. [26] fine motor development at 24 months discriminated EL-Autism from EL-TD and EL-ASD only from TL. Moreover, the exploration of various subcomponents of fine motor development by Kaur et al. [43] during a play situation showed less grasping and dropping of a rigid ball, less mouthing of a rattle at 6 months, together with delayed dropping of objects between 12 and 15 months, in EL compared to TL. Conversely, Sacrey et al. [49] and LeBarton et al. [64] did not find significant between-group differences on grasping.

#### Posture (*n* = 1)

Nickel et al. [27] explored the posture repertoire in EL and TL during everyday activities and semi-structured play, which were videotaped during several moments of development (between 6 and 14 months) and coded within the categories of lying, sitting, kneeling and standing. Between-group differences were found both in the variety of posture repertoire and in the stability over time of each posture. Between 6 and 12 months, EL-Autism were seen in half as many different postures as EL-TD and TL but this difference was no longer visible at 14 months. Moreover, at 6 months EL spent significantly more time in supported sitting and less time in unsupported sitting than TL. Finally, EL-Autism initiated new postures less frequently than EL-TD at 6, 9 and 12 months. However, by 14 months frequencies of infant-initiated postures for EL-Autism infants were much closer to the EL group mean.

### Repetitive/stereotyped behaviour (*n* = 5; Table 6)

This domain refers to repetitive and/or stereotyped behaviour, body movements, interests and use and manipulation of objects. Various assessment tools were chosen in different studies: ADOS [66], the Repetitive and Stereotyped Movement Scales (RSMS; [73]), from which object and body scores were derived, and a report of parents’ concerns, developed by Sacrey et al. [19]. Parents of EL-ASD first noticed a peculiar pattern of repetitive-restricted behaviour (RRBs) at 9 months compared to parents of TL and at 18 months compared to parents of EL-TD [19]. Coherently, Chawarska et al. [50] found that repetitive behaviour at 18 months predicted an ASD diagnosis at 36 months. Elison et al. [51] found that repetitive use and manipulation of objects discriminated EL and TL at 12 months, while Damiano et al. [52] found higher rates of total RSMS in EL than TL at 12–24 months, but no interaction between group and RSM type. In both studies, EL-ASD and EL-TD did not significantly differ in the object cluster subscale. Repetitive body movements were explored in two studies: while Elison et al. [51] found a clear pattern of repetitive body movements in EL-ASD at 12 months compared to EL-TD and TL, Damiano et al. [52] found no clear difference between EL-ASD and EL-TD in the body movement repertoire. Finally, repetitive interests seem to better discriminate EL-ASD and EL-TD at an early stage (6–12 months) [46].

### Sensory processing (*n* = 5; Table 7)

Five studies explored the association between early differences in the sensory domain and later ASD diagnosis. Germani et al. [44] referred to the Infant Toddler Sensory Profile (ITSP; [70]), a parent-report measure of behavioural responses to sensory stimuli, across five sensory domains (auditory, visual, tactile, vestibular and oral) as well as of a child’s reaction to sensory experiences. Brian et al. [46] and Zwaigenbaum et al. [33] focused on atypical sensory behaviours and interests (i.e. use of play materials in a self-stimulatory way) observed during the administration of the AOSI [65], Wolff et al. [53] used the Sensory Experiences Questionnaire (SEQ; [74]), a parent-report measure of behavioural responses to a range of common sensory stimuli and Sacrey et al. [19] based their assessment on reports of parent’s sensory concerns from an interview that they had developed. Sacrey et al. [19] reported parents’ first concerns in sounds, texture and visual inspection in EL-ASD by 6 months compared to parents of EL-TD and TL (who did not differ) while Wolff et al. [53] reported parents’ concerns from 12 months, age at which parents of EL-ASD observed higher tactile and hyper-sensory responsivity, as compared to parents of EL-TD and TL, with differences increasing from 12 to 24 months in all sensory domains. Zwaigenbaum et al. [33] stated that the use of parts of the body or play materials in stereotyped, self-stimulatory ways (i.e. rubbing hands repeatedly over tables, dangling a string of beads and waving them in front of his/her eyes) at 12 months but not at 6 months predicted ASD at 24 months. Brian et al. [46] found that at 18 months both EL-ASD and EL-TD showed more atypical sensory behaviours (i.e. smelling of toys, staring at hands/shapes/objects, or feeling textures) compared to TL, while EL-ASD and EL-TD did not differ after Bonferroni’s correction. Finally, conflicting results emerge at the 24 months assessment. Germani et al. [44] found that EL-ASD showed abnormalities in the auditory processing compared to EL-TD and TL while no difference was found in the visual, tactile, vestibular and oral domains. On the contrary, Wolff et al. [53] reported anomalies in all sensory subdomains.

### Play (*n* = 2; Table 8)

Children’s play behaviour was explored through a parent questionnaire, developed by the authors [19], to deepen general parent concerns about play as well as a free play situation videotaped and coded [54]. The free play assessment explored various aspects of play: functional play (i.e. the appropriate use of an object or the conventional association of two or more objects), symbolic play (i.e. the ability to pretend an object is present when it is not or to extend the function of an object to another object), repeated play (i.e. repeated behaviour, repetition of functional or symbolic actions, atypical actions).

Parents of EL-ASD reported their first concerns about play skills at 9 months compared to parents of EL-TD and TL, who did not differ, after post-hoc analyses [19]. Results of the free-play assessment showed that at 18 months EL-ASD had significantly fewer novel self-directed and other-directed functional play behaviour (defined as the appropriate use of an object or the conventional association of two or more objects) than TL (although the differences in the self-directed functional play disappeared after controlling for verbal mental age). Furthermore, both EL-ASD and EL-TD exhibited greater levels of non-functional repeated play than TL (although this effect dropped out when controlling for verbal age), while no significant difference was found within the EL group. On the contrary, EL-ASD and TL did not differ in functional repeated play and in symbolic play [54].

### Temperament (*n* = 6; Table 9)

Several temperament dimensions have been explored in children at high risk for ASD. Results are based on the AOSI [65] in one study [46] and on the parent questionnaires Carey Temperament Scale (CTS; [75]), Toddler Behaviour Assessment Questionnaire (TBAQ; [76]), The Early Childhood Behaviour Questionnaire [77] and IBQ [67] in all others [33, 35, 55–57].

Brian et al. [46] assessed reactivity and transition from the AOSI [65]. Reactivity codes for the behavioural responses to objects and events and is expressed by under-reactivity (passivity) or over-reactivity while transition refers to the ease and consistency with which objects are withdrawn or to move from an activity to another, expressed as ‘inflexible adherence to routines’ in the DSM-5 [1]. Over-reactivity, under-reactivity and transition at 18 months discriminated EL-ASD from EL-TD and TL, as well as EL-TD from TL, and predicted ASD diagnosis at 36 months.

In parent questionnaires parents were asked about their child’s surgency, positive affect, approach, activity, adaptability, effortful control and emotion regulation.

Parents identified abnormalities already during the first year and beginning of the second year. According to del Rosario et al. [56] through the CTS parents reported lower levels of approach (which stands for the tendency to approach new situations and people) at 6 months, of adaptability (as slowness to change behaviour in meeting the expectations of others) at 6 and 12 months, in EL-ASD than EL-TD. The trend changed across time with EL-ASD having higher score than EL-TD at 24 and 36 months in both approach and adaptability scales [56]. EL-ASD presented a less active behaviour (i.e. child is mainly engaged in many quieter pursuits) than EL-TD at 6 and 12 months, but differences were no longer significant across later development [56]. Similarly, in Zwaigenbaum et al. [33] through the IBQ and TBAQ parents described EL-ASD as having lower activity levels at 6 months than EL-TD and TL. Pijl et al. [35] confirmed above results, as they found through the ICBQ and ECBQ diverging levels of surgency (referring to engaging with environment, approach behaviours, positive affect and activity levels) from 8 to 14 months in EL-ASD as compared to EL-TD. Additionally, analyses from the TBAQ-R and the IBQ-R revealed that EL-ASD showed less effortful control (as the (in)ability to inhibit a dominant response) than EL-TD from 14 months, according to Pijl et al. [35], but not according to Garon et al. [57].

Zwaigenbaum et al. [27] reported parents’ description of their EL-ASD children at 12 months as having more frequent and intense distress reactions (suggesting effortful attempts and difficulties to suppress emotions), less inhibitory control, less positive anticipation and affective responses than EL-TD and TL children. Lower positive affect at 12 months and effortful control at 24 months predicted ASD symptoms at 36 months [55].

Moving to the results from the 24-months-assessment, Garon et al. [57] found fewer approach behaviours at 24 months in EL-ASD than EL-TD and TL although normative levels at 24 months and 36 months were found by del Rosario et al. [56]. This trend of early impairment followed by later normalization was also found in del Rosario et al. [56] on the activity and adaptability scales. Finally, Garon et al. [57] reported higher scores on effortful emotion regulation at 24 months in EL than TL, while the two EL subgroups did not significantly differ.

## Discussion

The present paper provides an overview of the early non-social behavioural indicators of ASD. The search was restricted to studies that allocated children at high risk for ASD in one of two classes of developmental outcome: diagnosis of ASD and typical development. In addition, differences between EL and TL children, as well as between EL-ASD and EL children with developmental delay (EL-Other), are reported. We were particularly keen on exploring the developmental moment in which the differences among groups are first exhibited. Findings are clear for some domains while still conflicting for others. We described a characteristic as impaired in EL-ASD, when concordant results came from the majority of the studies assessing that domain, and always at least two studies, coming from different research groups. Results that are based on a single study should be interpreted with greater caution.

### Earliest non-social precursors of ASD: attention disengagement, fine and gross motor milestones, repetitive/stereotyped behaviour

There is sufficient evidence in the review to state that EL children with later ASD are characterised by early impairments in attention disengagement, in gross and fine motor development, as well as characteristic restricted and repetitive interests and behaviours and atypical sensory experiences, compared to EL children with a typical developmental outcome. This pattern discriminates EL from TL children already between 6 and 12 months. From the 12th month and throughout the second year the aforementioned pattern differentiates between EL-ASD and EL-TD. Moreover, early attention disengagement appears to have a predictive role towards later ASD diagnosis, as documented by three studies [31–33]. The same conclusion was reached by single studies for several other domains (visual search, visual tracking, motor control, repetitive behaviour, sensory processing, temperament), but these findings need to be replicated.

#### Attention

Different theoretical models offer alternative interpretations of the results on attention disengagement. Landry and Bryson [15], for example, adduced to the potential role of domain-general processes in producing some of the core features of autism. According to this hypothesis, attention disengagement is a basic component of the orienting network, and is supposed to be important for the regulation of emotional states and for an appropriate social interaction. Analogue connection is present at an anatomic level, as the orienting network is closely connected to the arousal or vigilance network [78]. Thus, a dysfunction in a basic process—such as attention disengagement—might underlie some of the core symptoms of ASD, including the social interaction and communication deficit, since orienting towards another stimulus (in this case another person) is a prerequisite for it. Alternatively, Bedford et al. [31] interpreted the simultaneous presence of attention disengagement and gaze following in EL-ASD at 13 months in light of a cascade (or additive) risk model, suggesting that measures of social and non-social attention contribute to ASD outcome via separate pathways. Contrary to disengagement, attention shift and engagement seem to be preserved in EL children [32, 33, 59].

If we consider disengagement, shift and engagement as three interrelated, but independent subcomponents of visual orienting, attention disengagement serves to relieve the visual system and allows it to reorient towards a salient target in the visual scene. While reaction time to disengage seems longer in EL-ASD, it might not affect the ability to orient towards a non-social stimulus, which seems to be preserved. Results in this direction have been confirmed by other studies [32, 51]. On the contrary, several studies that found deficits in both attention disengagement and shift in children with ASD did not provide an independent measure of the two operations [60, 79–81].

Alternatively, we might adduce the absence of significant results in the present review to the small sample sizes [29, 62]. Noteworthy is the finding of Sacrey et al. [62] in which EL-ASD at 12 months were more likely to maintain their gaze on the toy after it was grasped, as well as to disengage and re-engage the toy prior to grasping, compared to EL-TD and TL, although differences were no longer visible at 36 months. This over-focus of attention, together with the tendency to re-engage the same stimulus, have often been documented [14, 82, 83] both in the social and non-social domain, and they have been explained as a deficit in redirecting attention towards new stimuli, once their attention is engaged.

To sum up “the reduced ability to disengage attention from a stimulus (after previously engaging it) is one of the most consistently found cognitive deficits in people with ASD from infancy onwards” [84].

#### Gross and fine motor milestones

Delays and atypicalities in motor milestones have been a recurrent finding in infant studies on ASD and were already recognized by Kanner [85] and Asperger [86]. Unlike the literature on attention disengagement, opposite results have also been documented [87]. However, and compared to Ozonoff et al. [87], we attempted to give a closer focus on the developing motor system by highlighting several subdomains. According to the present review, at 6 months the Fine motor scale discriminated EL versus TL, but no differences within the EL group could be identified [26, 41, 42, 47]. The Gross motor scale did not discriminate EL from TL children during the first year [42, 64] and in two studies a TL group was missing [41, 47]. Noteworthy, Estes et al. [26] reported that a subgroup of EL children with later diagnosis of autism (EL-Autism) showed impaired gross motor development already at 6 months, compared to TL. It is from the second year that EL-ASD showed lower scores than EL-TD in Gross and Fine motor scales [41, 42, 47]. These data are particularly informative of the developmental trajectories of EL-ASD children. While early gross motor milestones (i.e. walking) are generally acquired at expected ages in EL children, more complex and fine motor skills hardly emerge. Accordingly, we can hypothesize a developmental decline between 14 and 24 months, a period in which EL-ASD children might be particularly vulnerable. A possible explanation was proposed by Akshoomoff et al. [88]. They stated that the brain volume of young EL-ASD is likely to begin in the typical range, and becomes larger than that of controls as early as at 6 months [89], but certainly by 2–4 years [90]. Wolff et al. [91] found higher fractional anisotropy of white preceding matter tract in EL-ASD than EL-TD from 6 months until 24 months. It is noteworthy that the onset of white matter tract differences is consistent with the differences in gross motor development, both observed at 6 months. This process of pejorative neurobehavioural alteration starts very early and might explain the finding of Estes et al. [26] of impaired gross motor development of EL children with an autism outcome already at 6 months, with earlier and more severe deficits in children whose ASD symptoms are later more severe.

#### Restricted and repetitive behaviours

Results on this domain are particularly promising, as EL-ASD showed atypical repetitive interests and body movements compared to EL-TD already in the first year (6–12 months, Brian et al. [46], 12 months, Elison et al. [51]) and repetitive/stereotyped behaviours from 18 months, emerging coherently from parent reports [19] and standardized instruments within laboratory conditions [50].

The present results are in line with the acknowledgement of restricted and repetitive behaviours as a core symptom of ASD [1, 92]. On the contrary, the question of why repetitive and restricted behaviours are consistent in people with ASD has received far less attention. In their systematic review, Leekam et al. [25] attempted to summarize the literature of the last 15 years on the neurobiological, developmental and cognitive influences that evoke RRBs. Among the potential candidate factors, a neurobiological explanation [93, 94] proposes that RRBs are the outcome of genetic vulnerability (chromosomal mutations). Support for genetic involvement in RRBs has been confirmed by more recent reviews [95–97]. Additionally, RRBs have proved to be an outcome of experiential deprivation or restriction [23]. Finally, both genetic and environmental evidence comes from mouse models and should be confirmed in studies on human populations.

Neuropsychological approaches propose a connection between executive functions and RRBs [98, 99]. However, the nature of the association between cognitive dysfunctioning and repetitive behaviour misses some clear evidence, due to the mixed nature of the results. Finally, the developmental psychology model (first proposed by Thelen [100]) postulates that RRBs are immature behavioural responses that are normal in young children with a typical development but restricted to an early period in children’s lives, as they come increasingly under voluntary control as infants begin to develop goal-directed actions. Possible triggers for RRBs in ASD have also been identified in emotional and motivational states [101, 102].

#### Relation between deficit in attention, motor milestones and repetitive/stereotyped behaviour

Little is known about the relationship between attention and motor deficits in ASD. Ravizza et al. [103] described three alternative models: the resource allocation model assumes that attention impairment reduces other mental resources, producing impairments in other, non-affected domains (i.e. motor domain). The shared process model hypothesizes an impairment in a shared process, common to attention and motor domains. The independent account model assumes the absence of a core impairment able to explain both attention and motor deficits. Ravizza et al. [103] found that orienting attention was related to motor performance. However, the degree to which participants improved on the attention task when the motor demands were reduced was unrelated to the participants’ performance on the motor task. Indeed, a shared process model seems to better explain the findings, compared to a resource allocation process. Confirmations come from identified anatomical abnormalities. Parietal lobe and the cerebellum are both associated with attention and motor coordination, and seem to be implicated in the neuropathology associated with ASD [104]. Furthermore, the authors found a significant correlation between the severity of stereotyped movements and motor control, while no direct link was found between orienting attention and RRBs.

### Preliminary results from other non-social behaviours: sensory atypicalities, social attention, play and temperament

The present review leaves some open questions. No final conclusion was reached within the sensory domain, due to the heterogeneity of the sensory subcomponents assessed in each study. Similarly, the mentioned studies on temperament, although promising, need to be confirmed. Furthermore, the well-documented deficit in processing social stimuli in ASD was disconfirmed in the present review. Consequently, more research is needed to deepen alternative theoretical models. Finally, no conclusion about early atypical play behaviour could be achieved.

#### Sensory domain

Parents’ concerns towards atypical sensory interests in EL-ASD have been documented from 6 months on by Sacrey et al. [19], compared to parents of EL-TD and TL (who did not differ). Similarly, the use of body and play materials in a self-stimulatory way at 12 months was predictive towards ASD [33]. In Germani et al. [44] sensory abnormalities at 24 months in EL-ASD were limited to the auditory domain, while Wolff et al. [53] extended them to all sensory domains. These results might suggest that sensory atypicalities might be a specific trait for children who develop ASD not seen at a sub-threshold level.

However, opposite results were presented by Brian et al. [72] in which EL-ASD and EL-TD did not differ in atypical sensory behaviour at any time point, while sensory behaviours were able to discriminate EL versus TL at 18 months. In line with this finding, Watson et al. [105] found that EL children with later developmental atypicalities but not ASD were rated higher by their parents on the sensory processing cluster compared to children with typical development.

The inconclusiveness of these results, with the exception of anomalies within the auditory domain reported by two studies, has also been documented elsewhere. In a recent review by Johnson et al. [106] sensory atypicalities were documented in 90% of children who already received a diagnosis of ASD. On the contrary, the sensory domain is still understudied in young children at risk for the disorder.

A possible explanation might be the difficulty in characterising sensory difficulties in a strongly empirical manner, compared to more apparent social and cognitive symptoms. Coherently, although the unusual sensory responses have been noted since the first clinical descriptions of autism, they have been included in the diagnostic criteria for the disorder only very recently [1].

#### Social attention

Results from studies on face processing without social interaction interestingly show that EL fixate faces longer than TL [20, 37], or have a preference for local elements when processing visual stimuli (both social and non-social [107, 108]), findings that are possibly consistent with an emerging overly focal attention style. It should however be noted that only in one study this finding—prolonged looking time at faces—was actually related to the ASD outcome [37]. More studies should explore other measures of visual behaviour to test the hypothesis of a specific visual deficit towards social stimuli in children who later develop ASD. If replicated, the findings presented above would contradict models on ASD which assume less engagement with faces in ASD [109], but are consistent with Falck-Ytter et al. [40] and a growing body of research stating the absence of a deficit in early orienting specific to social stimuli (i.e. people, faces [109, 110]).

#### Play

Parents of EL-ASD reported atypical play behaviour at 9 months compared to EL-TD and TL [19]. This result is consistent with previous findings of play impairments early in development [111, 112] and suggests that children with later ASD interact with and explore the environment in a way that is atypical very early during development.

Although further replication is needed, these and other results argue for a renewed attention to the content and timing of parental concerns.

#### Temperament

Put together, studies reporting parents’ description outlined an early temperament profile of EL-ASD characterised by difficulties in approaching new people and situations, in changing behaviour according to people’s expectations, lower levels of activity, already at 6 months, compared to EL-TD. This pattern was accompanied by more frequent and intense distress reactions, less positive affect and less inhibitory control at 12 months. Similar findings have been documented by Visser et al. [113] in their systematic review. Altogether, results push for a closer focus on temperament subcomponents, considering that the actual research on temperament in pre-school years is virtually non-existent and limited by the retrospective nature of most observations.

#### Relationship between motor development and play

While the review suggests the importance to look at motor skills early during child development of EL children, fundamental motor skills are also crucial for the future development of efficient active play. Research has demonstrated that proficiency in fundamental motor skills is positively associated with physical activity, which is expressed in the form of active play in young children. With movement being a core element of play, the motor delay experienced in children with later diagnosis of ASD might have negative consequences for play in this population. More in detail, it has been hypothesized that ASD children are not physically able to engage in active play, due in part to their poor motor skills [114]. Structural and functional neuroimaging studies confirm such a close connection. In particular, they have shown that the cerebellum is a key structure, not only for sensorimotor control, but also for higher level functions, such as cognition and emotions, all three being relevant in play behaviour through the connections with cortical (premotor, prefrontal and posterior parietal) areas, and that various cerebellar dysfunctions are highly correlated with deficits in sensorimotor play, repetitive behaviours, cognition and affect in ASD [115]. In line with above, a few studies have explored the effects of motor skills’ interventions on adaptive skills, including object manipulation and object control during play [116, 117]. The inconsistency of the results drives for a further investigation with larger samples and longer follows-up to better understand the impact of the intervention, and its intensity, on play skills retention.

#### Relationship between temperament and sensory abnormalities

Of particular interest is also the potential association between temperament and sensory features in ASD. Brock et al. [118] linked extreme sensory features to lower levels of approach and higher levels of distractibility. First, more extreme sensory features were linked to reduced approach towards novel physical or social events. For instance, a child with ASD who is hyporesponsive, may not attend to a new sensory stimulus in the environment and would be less likely to approach that stimulus. On the other hand, a child with ASD who is hyperresponsive might approach less to avoid an aversive sensory response. Although distractibility was not included in the present review as a temperament subcomponent, it has been deepened within the attention domain. Brock et al. [118] found that hyporesponsiveness was most associated with distractibility. One explanation is that children who are less responsive to sensory stimuli may simply be more difficult to distract. Alternatively, some children with ASD who are hyporesponsive may be difficult to engage at all, or may be overfocused on irrelevant stimuli and have trouble disengaging attention [31].

Finally, it should be noted that visual search, motor control and atypical motor behaviour fell within the early signs of ASD, but results were based on a single study, for each domain, thus preventing strong conclusions to be reached.

### General discussion: delineating the non-social behavioural profile of EL-ASD

For the sake of readability, results from each non-social domain have been presented independently. Now, summing up, the review offers enough elements to delineate a profile of an EL child that could possibly receive a diagnosis of ASD at 36 months or later. Manifestations and time of symptoms’ appearance are clear for some domains, but are uncertain for others.

Preliminary results based on single studies reported that parents described EL-ASD between 6 and 12 months as having a peculiar temperamental profile, atypical play skills as well as sensory abnormalities. Similarly, different assessment methods showed stereotypic interests in EL-ASD at the same time point.

A replicated, more robust finding is a temporal overlap in the appearance of some attention and motor deficits in EL children during the first year and in EL-ASD from 12 months on. Slower disengagement of attention and less mature fine motor behaviours clearly characterised EL as compared to TL between 6 and 12 months. By the end of the first year and during the second year EL-ASD showed atypical attention disengagement, abnormal fine and gross motor development, that discriminate them from EL-TD. Functional neuroimaging studies stated that abnormal integrity in the white matter in several brain regions has been found in young children with autism. It has been hypothesized that age, and thus the timing of white matter disruptions in the autistic brain, is particularly relevant [119, 120]. Recent findings led to hypothesize that the last part of the first year of life could be a crucial time for anatomical changes in the brain of children who will later be diagnosed with ASD [121]. The concurrent timing with the ASD symptoms’ onset suggests that brain changes from six months on may have an important role to the pathogenesis of autism behaviour [87] and could also explain the first months of relatively typical postnatal development in ASD. In addition, atypical patterns of connectivity are not specific to any single brain region, or behavioural domain, thus offering a potential explanation of the simultaneous appearance of multiple abnormalities at once. Overall, these data suggest the existence of a period of critical importance to the pathogenesis of ASD and reinforce the importance of a developmental approach to brain and behavioural changes during this time.

## Implications for clinical practice

Put together, these findings show the progress made towards an earlier referral of ASD. Accordingly, they also offer new challenges to clinical practice.

Over the past two decades, ASD diagnostic instruments have been refined to offer a valuable source of information about a child that can help clinicians make better-informed judgments. Among them, the Autism Diagnostic Interview-Revised (ADI-R; [122]) and the Autism Diagnostic Observation Schedule 2 (ADOS-2; [123]) were developed to operationalize the core symptoms of ASD. These measures provide a standardized observation of current social-communicative behaviour, with excellent inter-rater reliability, internal consistency and test–retest reliability [66, 124]. However, an expert clinical view has shown to be more accurate than the use of standardised assessment instruments and the strict application of diagnostic criteria [123].

In line with the present findings, a piece of the puzzle seems to be missing from the current ASD diagnostic indications. Firstly, not only social-communication deficits, but also motor and attention abnormalities systematically characterize EL-ASD children compared to EL-TD, by the time in which the clinical diagnosis is applied. This finding should result in a new clinical approach to ASD, in which, through renewed diagnostic instruments, expert clinical judgment encompasses cognitive, motor and social anomalies.

Secondly, non-social abnormalities appear much before social impairments are fully manifest. Indeed, attention and motor deficits discriminate EL from TL from 6 months on, in line with parents’ reports but also empirical screening measures. This result suggests the need for a new attitude towards the identification of ASD, in which a careful expert eye will recognise atypicalities in the first stages of cognitive and motor development. On the contrary, the absence of early social deficits cannot guarantee the exclusion of the risk for the later appearance of the clinical condition. Coherently, there seems to be some evidence that screening for ASD in children where an early developmental concern was already identified, may result in better sensitivity and specificity [125, 126].

Thirdly, the ability of non-social signs to discriminate between groups increases with age. From 12 months EL-ASD show distinct attention and motor impairments, compared to EL-TD. This last finding suggests that, rather than being indicators of general developmental delay, non-social impairments are specific for ASD.

Overall, the present findings urge for a multidisciplinary approach to the diagnostic assessment. Primary healthcare practitioners should directly elicit and note examples of characteristic social and non-social behaviours from parental report and where possible from direct observations. Parents’ early concerns about motor development, distractibility, repetitive interests and behaviours should be considered as possible indicators of ASD, that warrant further monitoring. If these behaviours persist, then referral to a child development specialist for further assessment is strongly advised. In this context, screening should be implemented at regular intervals, ideally from children’s first birthday [127].

Finally, equally relevant are the implications for therapeutic interventions. Several studies have shown that targeted interventions improve the outcome of children with ASD (see Dawson and Burner [128], LeBlank and Gillis [129], Zwaigenbaum et al. [130] for reviews). The co-presence of such diverse symptoms would recall for a combination of targeted interventions, intended to retrieve each impaired domain. Moreover, the enactment of early intervention would be a natural prosecution of the clinical profile emerged from these data. Despite the absence of direct empirical evidence that early compared to late interventions have a specific advantage, general consensus exists on the concept of early interventions supported by developmental principles.

## Limitations and directions for future research

The absence of frequent repeated assessments of non-social precursors, with many studies not including any evaluation between 6 and 12 months, or between 12 and 24 months, represents a potential limit of the present review. Additionally, we report studies in which ASD diagnosis is based exclusively on instruments that identify socio-communicative deficits and repetitive behaviours, but leave several domains underexplored. Furthermore, the studies do not follow children up after their third birthday and therefore give no information on the stability of the diagnosis.

The acknowledgement of the increased prevalence of ASD in siblings of individuals with the same diagnosis represents an important facilitation for the systematic study of this clinical condition. The creation of databases of EL of ASD families in each country would facilitate the recruitment of participants for future research and favour the implementation of bigger surveys.

Overall, the finding of early detection of non-social symptoms and their stability across time suggests the need to systematically search for precursors long before the diagnosis can be implemented. Consequently, ASD should be reconsidered as an impairment of neurodevelopment that emerges gradually. Underdeveloped motor milestones, systematic difficulties in directing attention towards new stimuli and repetitive and stereotyped interests and behaviours are red flags for autism and should be addressed as specific precursors of ASD.

Parents’ descriptions of everyday activities and observations of interactions with peers should be maintained in future studies because they uncover singular behaviours and atypical reactions to changes in the environment that are meaningful for characterising the ASD spectrum.

Finally, future studies will need to assess if and how the various non-social behaviours are interrelated and their connections at a neurocognitive level. A more comprehensive vision of early ASD precursors could result from the inclusion of early social and non-social precursors as well as its reciprocal relations.

## Electronic supplementary material

Below is the link to the electronic supplementary material.

Supplementary file1 (DOCX 970 kb)
