# Supplementary material for: Early non-social behavioural indicators of autism spectrum disorder (ASD) in siblings at elevated likelihood for ASD: a systematic review
Source: Eur Child Adolesc Psychiatry. 2020 Feb 22;30(4):497–538. doi: 10.1007/s00787-020-01487-7 (PMC8041710; doi:10.1007/s00787-020-01487-7)
Supplement: Supplementary file 1 — Supplementary file1 (DOCX 970 kb) [file 787_2020_1487_MOESM1_ESM.docx]

| **Table** Risk of bias assessment (Newcastle–Ottawa Quality Assessment Scale criteria) | | | | | | | | | |
| --- | --- | --- | --- | --- | --- | --- | --- | --- | --- |
| **Selection** | | | | | **Comparability** | **Outcome** | | | **Quality score** |
| **Study** | **Representativeness of Exposed Cohort** | **Selection of Non-Exposed Cohort from Same Source as Exposed Cohort** | **Outcome of interest was not present at start of study** | **Ascertainment of exposure** | **Comparability of cohorts** | **Assessment of outcome** | **Follow-up long enough** | **Adequacy of follow-up of cohorts** |  |
| Bedford et al., 2012 | Not representative | yes | yes | Siblings’ diagnosis was confirmed by expert clinical judgment using the Development and Wellbeing Assessment (DAWBA) and the parent-report Social Communication Questionnaire (SCQ) | The Mullen Scales of Early Learning (MSEL) composite standard score, as indicator of developmental quotient, was included as a covariate, to account for any group differences in IQ | Autism Diagnostic Observation Schedule-Generic (ADOS-G), Autism Diagnostic Interview-Revised (ADI-R), expert clinical judgment according to ICD-10 | yes | 73 (35 EL and 38 TL) out of 104 (54 EL, 50 TL) participants completed the visit at 7 and 13 months | Good |
| Bedford et al., 2014 | Not representative | yes | yes | Siblings’ diagnosis was confirmed by expert clinical judgment using the DAWBA and the SCQ | The MSEL composite standard score at 13 months was included as a covariate, to account for any group differences in IQ | ADOS-G, ADI-R, expert clinical judgment according to ICD-10 | yes | 53 out of 54 EL and 48 out of 50 TL were included in the 36 months visit | Good |
| Brian et al., 2008 | Not representative | yes | yes | Siblings’ diagnosis was confirmed by expert clinical judgment through a clinical interview using DSM-IV criteria, and by the administration of the Autism Diagnostic Observation Schedule (ADOS) | Groups at 18m were matched on age, not on developmental level and socio-economic status | ADOS-G, ADI-R, expert clinical judgment according to DSM-IV-TR | yes | 228 (155 EL, 73 TL) participants were followed-up until 36 months.  No information on the initial sample size was given | Good |
| Bryson et al., 2018 | Not representative | yes | yes | Siblings’ diagnosis was confirmed by expert clinical judgment through a clinical interview using DSM-IV criteria, and by the administration of the ADOS | The MSEL composite standard score was included as a covariate | ADOS-G, ADI-R, expert clinical judgment according to DSM-IV-TR | yes | 136 participants (83 EL and 53 TL) were followed-up until 36 months visit. No information on the initial sample size was given | Good |
| Chawarska et al., 2014 | Data on the source community were not clearly stated | no TL group was included | yes | Siblings’ diagnosis was confirmed by expert clinical judgment, ADOS-G and ADI-R | The MSEL subscales of Visual Reception and Receptive Language were included in the model as covariate | ADOS-G, expert clinical judgment according to DSM-5 | yes | 719 EL participants were included at 36 months assessment.  No TL group was included | Fair |
| Cheung et al., 2018 | Not representative | yes | yes | Siblings’ diagnosis was confirmed by expert clinical judgment using the DAWBA and the SCQ | The MSEL composite standard score, age and sex were entered into the model as covariates, and did not have a significant effect on between-group differences | ADOS-2, ADI-R, expert clinical judgment according to DSM-5 | yes | 140 out of 143 (116 EL and 27 TL) participants were followed-up until 36m visit. 3 EL did not take part in the 36m visit | Good |
| Choi et al., 2018 | Not representative | Not clearly stated.  Significant between-group difference in maternal education | yes | Siblings’ diagnosis was verified with ADOS, SCQ, PDDST-II or only specialised clinicians (for 5 participants) | Gender, socioeconomic status and the Visual Receptive score from the MSEL were included as covariates | ADOS | yes | 73 out 101 EL and 54 out of 69 TL were followed up until 36m | Fair |
| Christensen et al., 2010 | Not representative | yes | yes | Siblings’ diagnosis was confirmed by expert clinical judgment through a record review and, in cases where records were inconsistent, direct assessment using the ADOS | Verbal mental age (based on MSEL) at 18 months was included as covariate | MSEL, VABS, parent concerns’ interview | yes | 77 (58 EL, 19 TL) participants were followed-up until the 36 months visit.  No information on sample size at the beginning of the study is given | Good |
| Damiano et al., 2013 | Not representative | yes | yes | Siblings’ diagnosis was confirmed by expert clinical judgment, ADOS-G and ADI-R | Given between-group differences in maternal education, this variable was included as a covariate | ADOS, ADI-R, expert clinical judgment | yes | 40 (20 EL, 20 TL) participants were followed-up until the 36 months assessment.  No information on sample size at the beginning of the study is given | Good |
| de Klerk et al., 2014 | Not representative | yes | yes | Siblings’ diagnosis was confirmed by expert clinical judgment using the DAWBA and the SCQ | The MSEL composite standard score at 3 years was included as a covariate to account for any group differences in general intelligence | ADOS-G, ADI-R, expert clinical judgment according to ICD-10 | yes | 101 (53 EL and 48 TL) out of 104 (54 EL and 50 TL) participants were followed-up until 36 months. 44 (out of 53) EL and 40 (out of 50) TL provided valid data for the face recognition task at 36m | Good |
| del Rosario et al., 2014 | Not representative | yes | no TL group was included | Siblings’ diagnosis was confirmed by expert clinical judgment through a record review and, in cases where records were inconsistent, direct assessment using the ADOS | Gender was included in the analyses as covariate | ADOS-G, SCQ, expert clinical judgment according to DSM-IV-TR | yes | 37 EL were followed-up until 24m; 30 EL until 36 months.  No TL group was included in the study | Fair |
| Elison et al., 2014 | Not representative | yes | yes | Siblings had an ASD diagnosis, based on the scores on SCQ and ADI-R, conﬁrmed by medical records | Groups were matched on age, weeks of gestation, race/ethnicity, maternal education level, family income | ADOS-G, expert clinical judgment according to DSM-IV | yes | 158 (105 EL, 53 EL) participants were included in 36 months visit. One TL participant was excluded because he met ASD criteria.  No information on EL sample size at the beginning of the study is given | Good |
| Elsabbagh et al., 2011 | Not representative | no | yes | Siblings’ diagnosis was confirmed by expert clinical judgment using the DAWBA and the SCQ | Authors controlled for age at the first visit as a continuous variable | ADOS-G | yes | 27 out of 31 EL were included in the 36 months visit.  No TL group was included in the study | Fair |
| Elsabbagh et al., 2013 | Not representative | yes | yes | Siblings’ diagnosis was confirmed by expert clinical judgment using the DAWBA and the SCQ | Non-verbal T score (based on MSEL) and age were included as covariates | ADOS-G, ADI-R, expert clinical judgment according to ICD-10 | yes | 101 (53 EL and 48 TL) out of 104 (54 EL and 50 TL) participants were included in the 36 months visit | Good |
| Elsabbagh et al., 2013 | Not representative | yes | yes | Siblings’ diagnosis was confirmed by expert clinical judgment using the DAWBA and the SCQ | Infant’s age was included as a covariate in all analyses | ADOS-G, ADI-R, expert clinical judgment according to ICD-10 | yes | Data from 98 (52 EL and 46 TL) out of 104 (54 EL and 50 TL) participants were included in the 14m analyses and followed up until 36m | Good |
| Estes et al., 2015 | Not representative | yes | yes | Siblings’ diagnosis was based on scores on the SCQ and ADI-R, and was conﬁrmed by medical records | Three variables were included as covariate: site, mother education, differences between child’s actual age and age at first visit | ADOS, ADI-R, expert clinical judgment | yes | 308 (210 EL and 98 TL) out of 329 (222 EL and 107 TL) participants were followed-up until 36 months | Good |
| Falck-Ytter et al., 2018 | Not representative | yes | yes | Siblings’ diagnosis was confirmed by expert clinical judgment with inspection of the obtained medical records | Gender and the MSEL sub-scores for expressive and receptive language were included as covariate | ADOS-G/ADOS-2, ADI-R, expert clinical judgment according to DSM-5 | yes | 47 (33 EL, 14 TL) participants were followed-up until 36 months. 10 EL and 4 TL participants were excluded | Good |
| Gammer et al., 2015 | Not representative | yes | yes | Siblings’ diagnosis was confirmed by expert clinical judgment using the DAWBA and the SCQ | The verbal and non-verbal subscales of the MSEL were included as covariates to account for any group differences in general intelligence | ADI-R, Social Communication Questionnaire (SCQ), ADOS-G, expert clinical judgment according to ICD-10 | yes | 103 (53 EL and 50 TL) out of 104 (54 EL and 50 TL) participants were followed-up until 36 months visit | Good |
| Garon et al., 2009 | Not representative | yes | yes | Siblings’ diagnosis was confirmed by expert clinical judgment through a clinical interview using DSM-IV criteria, and by the administration of the ADOS | Gender and IQ were entered as covariates in a logistic regression analysis | ADOS-G,  ADI-R, MSEL, expert clinical judgment according to DSM-IV-TR | yes | 192 out of 211 (138 EL and 73 TL) participants were followed-up until 36 months. The authors do not specify the proportion of loss in EL and TL groups | Good |
| Garon et al., 2016 | Not representative | yes | yes | Siblings’ diagnosis was confirmed by expert clinical judgment through a clinical interview using DSM-IV criteria, and by the administration of the ADOS | The MSEL composite standard score at 12 months was included as covariate | ADOS, ADI-R, MSEL, expert clinical judgment according to DSM-IV-TR | yes | 534 (373 EL and 161 TL) out of 545 (383 EL and 162 TL) were followed-up until 36 months | Good |
| Germani et al., 2014 | Not representative | yes | yes | Siblings’ diagnosis was confirmed by expert clinical judgment through a clinical interview using DSM-IV criteria, and by the administration of the ADOS | Participants were matched on gender and age, but not on the general developmental level (MSEL) | ADOS, ADI-R, MSEL, expert clinical judgment according to DSM-IV-TR | yes | 91 (60 EL and 31 TL) participants were included in the last assessment at 36 months.  No information on the initial sample size is included | Good |
| Gliga et al., 2015 | Not representative | yes | yes | Siblings’ diagnosis was confirmed by expert clinical judgment using the DAWBA and the SCQ | Age and IQ were included as covariates | AOS (15m) ADOS-2(24m) | yes | 94 (69 EL and 25 TL) out of 109 (82 EL and 27 TL) participants took part in the 24 months assessment | Good |
| Iverson et al., 2019 | Not representative | yes | yes | Siblings’ diagnosis was confirmed using the ADOS, ADI-R scores, SCQ | Participants were comparable on ages at enrolment, first assessment and outcome assessment | ADOS, expert clinical judgment according to DSM-IV_TR | yes | 625 out of 648 participants were followed up until 36m.  No indication of the number of excluded/missing participants in each group was given | Good |
| Kaur et al., 2015 | Not representative | yes | yes | Siblings’ diagnosis was confirmed based on the ADI-R, expert clinical judgment, and/or medical record | Groups were matched on age, gender, ethnicity and socio-economic status | ASQ-3, 116 M-CHAT, 117 follow-up inquiries with parents | yes | 29 (14 EL and 15 TL) out of 32 (16 EL and 16 TL) participants were followed-up until 24m | Good |
| Landa et al., 2006 | Not representative | yes | yes | Siblings’ diagnosis was confirmed by access to medical record review and parental report | Between-group differences were controlled for age and socio-economic status | ADOS-G, expert clinical judgment according to DSM-IV | yes | 87 (60 EL and 27 TL) participants were followed-up until 24 months, as compared to 58 EL participants at 6 months.  No information on initial TL sample size is given | Good |
| Landa et al., 2012 | Not representative | yes | yes | Siblings’ diagnosis was confirmed by access to medical record review and parental report | Models were adjusted for non-verbal cognitive functioning (based on MSEL) | ADOS-G,  expert clinical judgment | yes | 225 (173 including EL-TD and TL and 23 EL-ASD) out of 235 (204 EL and 31 TL) participants were followed-up until 36 months evaluation.  No clear statement on participants’ loss in EL and TL group was made | Good |
| LeBarton et al., 2019 | Not representative | yes | yes | Siblings’ diagnosis was confirmed by expert clinical judgment using the ADOS | Gender, socio-economic status, age at time of motor assessment were included as covariate | ADOS-2 or ADOS-G, expert clinical judgment | yes | No information on EL and TL sample size at the beginning of the study is given | Good |
| Libertus et al., 2014 | Sources of recruitment are not stated | not clearly stated | yes | Confirmed ASD diagnosis | Between-group differences remained after controlling for non-verbal problem solving | ADOS-G,  expert clinical judgment according to DSM-IV | yes | 129 (107 EL and 22 TL) out of 143 (114 EL and 29 TL) participants were followed-up until the 36 months evaluation | Fair |
| Nickel et al., 2013 | Not representative | not clearly stated | yes | Siblings’ diagnosis was confirmed by expert clinical judgment using the ADOS | Parental level of education was comparable between groups | ADOS-G, expert clinical judgment according to DSM-IV | yes | 36 (18 EL and 18 TL) out of 40 (22 EL and 18 TL) participants were included in the follow-up at 14 months | Fair |
| Pijl et al., 2019 | Not representative | yes | yes | Siblings’ diagnosis was confirmed by expert clinicians using information from the DAWBA and the SCQ | Gender and age at first visit were included as covariates | ADOS-2, ADI-R, expert clinical judgment according to DSM-5 | yes | 199 (133 EL and 66 TL) out of 247 (170 EL and 77 TL) were followed up until 36m | Good |
| Rutherford et al., 2015 | Not representative | no | yes | Siblings’ diagnosis was confirmed by expert clinical judgment using the ADOS-G | Between-group differences were controlled for gender | ADOS-2 | yes | 62 (31 EL, 31 TL) participants were followed-up until 36 months.  No indication of initial sample size is given | Fair |
| Sacrey et al., 2013 | Not representative | yes | yes | Siblings’ diagnosis was confirmed by expert clinical judgment through a clinical interview using DSM-IV criteria, and by the administration of the ADOS | Gender was included as a covariate in all analyses | ADOS-G,ADI-R, expert clinical judgment according to DSM-IV-TR | yes | 30 participants were followed-up until 36 months.  None of the participant was lost in the follow-up | Good |
| Sacrey et al., 2015 | Not representative | yes | yes | Siblings’ diagnosis was confirmed by expert clinical judgment by a clinical interview using DSM-IV criteria, and by the administration of the ADOS | The MSEL and the Vineland Adapted behaviour scale (VABS) were assessed at each time point but it is not specified whether they are included as covariate. Age was included as covariate | ADOS-G, ADI-R, expert clinical judgment according to DSM-IV | yes | 237 (168 EL, 69 TL) out of 296 (217 EL, 79 TL) participants were followed-up until 36 months | Good |
| Sacrey et al., 2018 | Not representative | yes | yes | Siblings’ diagnosis was confirmed through the ADOS and expert clinical judgment according to DSM-IV-TR | Groups were comparable in age at first assessment | ADOS-G, ADI-R, expert clinical judgment according to DSM-IV-TR | yes | None of the participants was lost in the follow-up | Good |
| St. John et al. 2018 | Not representative | yes | yes | Siblings diagnosis was based on the SCQ and the ADI-R, and was conﬁrmed by medical records | Gross ad Fine motor subscales of the MSEL were added as covariate | ADOS-2, ADI-R, expert clinical judgment according to DSM-IV-TR | yes | 174 (125 EL, 49 TL) out of 262 (186 EL, 76 TL) participants were followed-up until 24 months | Good |
| Wass et al., 2015 | Not representative | yes | yes | Siblings’ diagnosis was confirmed by expert clinical judgment using the DAWBA and the SCQ | Age and general developmental level (MSEL) were included as covariate in all analyses | ADOS-G, ADI-R, expert clinical judgment according to ICD-10 | yes | 93 out of 94 (54 EL, 50 TL) participants were followed-up until 36 months | Good |
| Wolff et al., 2018 | Not representative | yes | yes | Siblings’ diagnosis was confirmed with the ADI-R and the SCQ | Sex, general developmental level (MSEL) and maternal education were included as covariates | ADOS, ADI-R, MSEL, expert clinical judgment according to DSM-IV-TR | yes | 263 out of 321 EL and 115 out of 135 TL were followed up until 24m | Good |
| Zwaigenbaum et al., 2005 | Not representative | yes | yes | Siblings’ diagnosis was confirmed by expert clinical judgment through a clinical interview using DSM-IV criteria, and by the administration of the ADOS | Groups were matched by gender and age | ADOS-G, ADI-R, expert clinical judgment according to DSM-IV | yes | 65 out of 150 EL participants were followed-up until the 24 months assessment.  No TL comparison group was included in the paper | Good |
|  |  |  |  |  |  |  |  |  |  |
